# Multi-scale pathology image texture signature is a prognostic factor for resectable lung adenocarcinoma: a multi-center, retrospective study

**DOI:** 10.1186/s12967-022-03777-x

**Published:** 2022-12-14

**Authors:** Yumeng Wang, Xipeng Pan, Huan Lin, Chu Han, Yajun An, Bingjiang Qiu, Zhengyun Feng, Xiaomei Huang, Zeyan Xu, Zhenwei Shi, Xin Chen, Bingbing Li, Lixu Yan, Cheng Lu, Zhenhui Li, Yanfen Cui, Zaiyi Liu, Zhenbing Liu

**Affiliations:** 1 School of Computer Science and Information Security, Guilin University of Electronic Technology, Guilin, 541004 China; 2 Department of Radiology, Guangdong Provincial People’s Hospital, Guangdong Academy of Medical Sciences, Guangzhou, 510080 China; 3 Guangdong Provincial Key Laboratory of Artificial Intelligence in Medical Image Analysis and Application, Guangdong Provincial People’s Hospital, Guangdong Academy of Medical Sciences, Guangzhou, 510080 China; 4 Guangdong Cardiovascular Institute, Guangzhou, 510080 China; 5 School of Medicine, South China University of Technology, Guangzhou, 510006 China; 6 Department of Radiology, Guangzhou First People’s Hospital, School of Medicine, South China University of Technology, Guangzhou, 510180 China; 7 Department of Pathology, Guangdong Provincial People’s Hospital Ganzhou Hospital (Ganzhou Municipal Hospital), 49 Dagong Road, Ganzhou, 341000 China; 8 Department of Pathology, Guangdong Provincial People’s Hospital, Guangdong Academy of Medical Sciences, Guangzhou, 510080 China; 9 Department of Radiology, The Third Affiliated Hospital of Kunming Medical University, Yunnan Cancer Hospital, Yunnan Cancer Center, Kunming, 650118 China; 10 Department of Radiology, Shanxi Province Cancer Hospital, Shanxi Hospital Affiliated to Cancer Hospital, Chinese Academy of Medical Sciences/Cancer Hospital Affiliated to Shanxi Medical University, Taiyuan, 030013 China

**Keywords:** Lung adenocarcinoma, Prognosis, Texture analysis, Whole slide image, Artificial intelligence

## Abstract

**Background:**

Tumor histomorphology analysis plays a crucial role in predicting the prognosis of resectable lung adenocarcinoma (LUAD). Computer-extracted image texture features have been previously shown to be correlated with outcome. However, a comprehensive, quantitative, and interpretable predictor remains to be developed.

**Methods:**

In this multi-center study, we included patients with resectable LUAD from four independent cohorts. An automated pipeline was designed for extracting texture features from the tumor region in hematoxylin and eosin (H&E)-stained whole slide images (WSIs) at multiple magnifications. A multi-scale pathology image texture signature (MPIS) was constructed with the discriminative texture features in terms of overall survival (OS) selected by the LASSO method. The prognostic value of MPIS for OS was evaluated through univariable and multivariable analysis in the discovery set (n = 111) and the three external validation sets (V_1_, n = 115; V_2_, n = 116; and V_3_, n = 246). We constructed a Cox proportional hazards model incorporating clinicopathological variables and MPIS to assess whether MPIS could improve prognostic stratification. We also performed histo-genomics analysis to explore the associations between texture features and biological pathways.

**Results:**

A set of eight texture features was selected to construct MPIS. In multivariable analysis, a higher MPIS was associated with significantly worse OS in the discovery set (HR 5.32, 95%CI 1.72–16.44; *P* = 0.0037) and the three external validation sets (V_1_: HR 2.63, 95%CI 1.10–6.29, *P* = 0.0292; V_2_: HR 2.99, 95%CI 1.34–6.66, *P* = 0.0075; V_3_: HR 1.93, 95%CI 1.15–3.23, *P* = 0.0125). The model that integrated clinicopathological variables and MPIS had better discrimination for OS compared to the clinicopathological variables-based model in the discovery set (C-index, 0.837 vs. 0.798) and the three external validation sets (V_1_: 0.704 vs. 0.679; V_2_: 0.728 vs. 0.666; V_3_: 0.696 vs. 0.669). Furthermore, the identified texture features were associated with biological pathways, such as cytokine activity, structural constituent of cytoskeleton, and extracellular matrix structural constituent.

**Conclusions:**

MPIS was an independent prognostic biomarker that was robust and interpretable. Integration of MPIS with clinicopathological variables improved prognostic stratification in resectable LUAD and might help enhance the quality of individualized postoperative care.

**Supplementary Information:**

The online version contains supplementary material available at 10.1186/s12967-022-03777-x.

## Background

Lung cancer is one of the most common malignant tumors worldwide, with the highest mortality rate [1, 2]. Lung adenocarcinoma (LUAD) is the most common subtype of lung cancer [3], accounting for 40% of all lung cancer types and more than 55% of non-small cell lung cancer. For patients with resectable LUAD, surgical resection with curative intent is the standard of care [4], but a significant portion of patients develop disease recurrence and die even after resection of the entire tumor mass [5]. Tumor-node-metastasis (TNM) stage [6] and tumor differentiation are traditionally considered to be the important postoperative prognostic factors, but significant differences in postoperative prognosis exist among LUAD patients with the same TNM stage and tumor differentiation due to tumor heterogeneity [7]. Therefore, a novel prognostic biomarker is needed to quantify the biological behavior of the tumor for precise risk stratification in resectable LUAD.

Histopathological slide, providing morphological information on tumors and their microenvironment at the tissue and cellular levels, is the gold standard for lung cancer diagnosis [8, 9]. Tumor development and growth depend highly on their interactions with the associated microenvironment [10]. Typically, pathologists visually examine Hematoxylin and Eosin (H&E)-stained slides from low to high magnification under a microscope to qualitatively assess the histopathological pattern of the tumor, which can help predict cancer behavior to a certain degree. Nevertheless, manual assessment is time-consuming and subjective. In addition, there are many sub-visual attributes of tumors in complex histopathological slides [11], allowing for a comprehensive characterization of the morphology of tumors and their microenvironment.

The rapid advancement of computer technology [12] and digital whole-slide images (WSIs) has opened up opportunities for identifying and quantifying sub-visual features correlated with prognosis. For example, texture features could quantitatively measure interactions between pixel intensities within a region of interest in an image. Recent studies also showed that image texture analysis plays an important role in quantifying underlying sub-visual tumor heterogeneity [13, 14]. However, these studies focused solely on single-scale image features, such as a single cell or tissue type, and ignored multi-scale information, which could diminish the accuracy of outcome prediction. Moreover, computer-extracted deep features from WSIs also appeared to be prognostic [15]. Nevertheless, deep learning models lack interpretability, and may have difficulties gaining widespread acceptance in clinical settings [16]. Thus, while previous studies have identified many prognostic biomarkers, there is still possible for improvement in terms of accuracy and interpretability.

In this study, we developed and validated a multi-scale pathology image texture signature (referred to as MPIS) using texture features at multiple magnifications extracted from digital H&E-stained WSIs, and then used MPIS in conjunction with Cox proportional hazards model to predict overall survival (OS) in patients with resectable LUAD. We hypothesized that MPIS was an independent prognostic factor for OS, and the integration of MPIS with clinicopathological variables would improve prognostic stratification in patients with resectable LUAD. Meanwhile, we also sought to demonstrate that the image-derived texture features correlated with the gene expression of biological pathways affecting tumor development.

## Methods

### Patients

This multi-center study was conducted using patients from four independent cohorts: a discovery set (Guangdong Provincial People's Hospital, GDPH) and three external validation sets (Yunnan Cancer Hospital, YNCH; Shanxi Provincial Cancer Hospital, SXCH; The Cancer Genome Atlas, TCGA). We enrolled LUAD patients who were treated with surgical therapy with curative intent at GDPH between 2007 and 2014, patients with resectable LUAD treated at YNCH from 2012 to 2014, and those treated at SXCH from 2014 to 2020. This study was approved by the Research Ethics Committee. Informed consent was waived because only retrospective imaging analysis was performed. Additionally, the TCGA dataset was downloaded from the Genomic Data Commons Data Portal (https://portal.gdc.cancer.gov/).

OS, defined as the time interval from surgery to death, was chosen as the endpoint event for our study. The baseline and clinicopathological variables were collected, including age at surgery, sex, smoking status, tumor site, adjuvant chemotherapy, differentiation, and TNM stage. We excluded the cases with treating with neoadjuvant therapy, remaining residual tumors, or dying within 1 month. The inclusion and exclusion criteria are detailed in Additional file 1: Section 1.

### Image acquisition

Digital WSIs were acquired from the H&E-stained diagnostic tissue slides of the primary tumor. The H&E-stained slides were scanned by Leica Aperio-AT2 USA scanner at 40 × magnification (0.252 μm/pixel). We controlled the image quality by excluding WSIs that were blurry, contained artifacts, exhibited poor staining, or lacked sufficient tumor tissues. In the TCGA dataset, some cases had multiple slides (one slide was selected for analysis according to image quality). Pathologists (BB Li with 5 years of clinical experience and LX Yan with 15 years of clinical experience) reviewed and agreed on the image quality for all WSIs. Additionally, these experienced pathologists annotated tumors and normal tissues on a set of 67 WSIs from GDPH for fine-tuning a pre-trained tumor segmentation model based on ResNet50 [17].

### Automatic tumor segmentation on WSIs

**Fig. 1:**
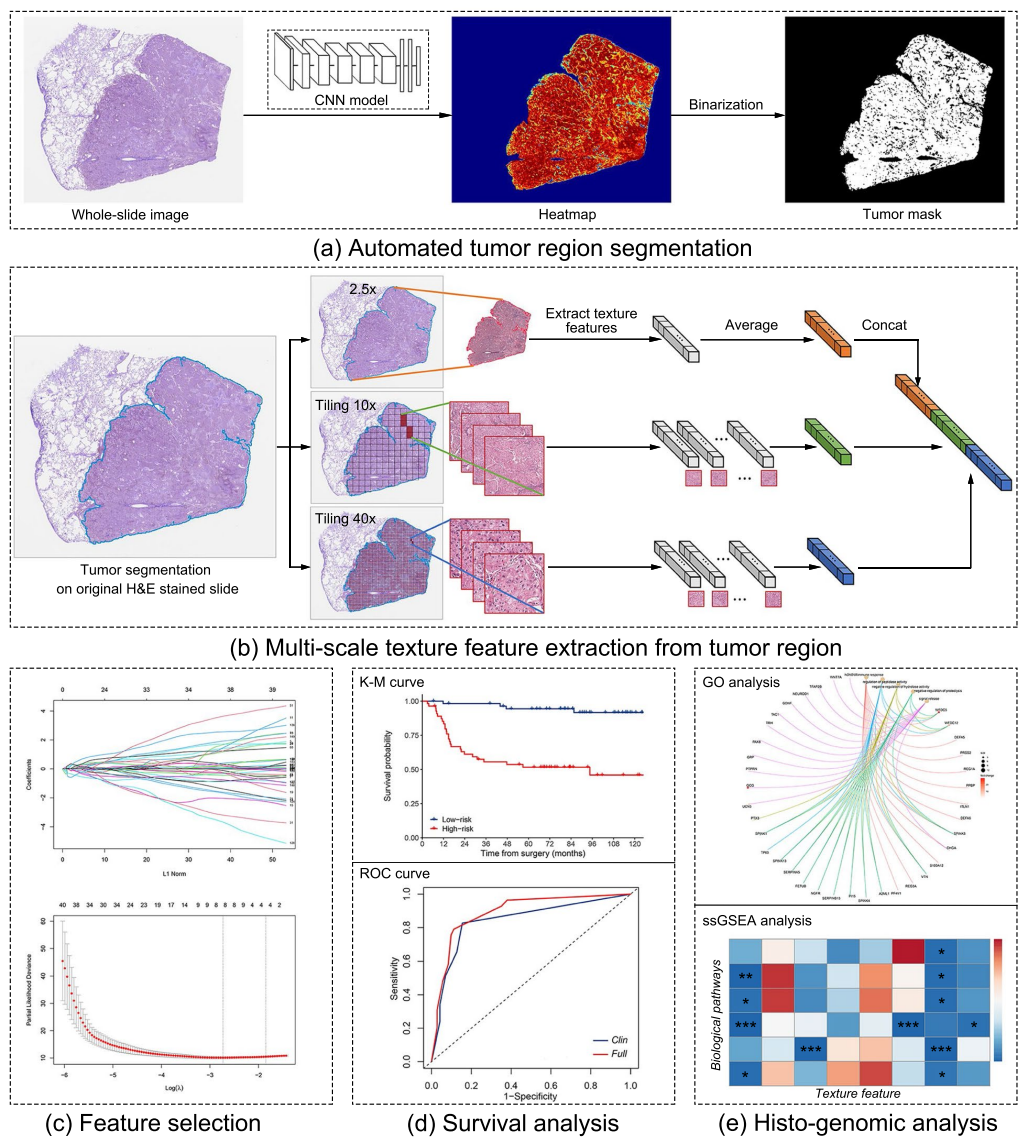
Overall workflow of this study. **a** Fully automated tumor region segmentation. **b** Image tile extraction from tumor region and multi-scale texture feature extraction, including 2.5 × , 10 × , and 40 × magnification. **c** Feature selection by the Lasso method. **d** Survival analysis and model development. **e** Histo-genomic analysis: the associated biological pathways are identified by gene ontology (GO) enrichment analysis and the relationships between the texture features and biological pathways are explored using single-sample gene set enrichment analysis (ssGSEA)

The overall workflow of this study is shown in Fig. 1. First, ResNet50 was employed to conduct tumor region segmentation. To reduce the amount of annotation, we used data from a similar domain for transfer learning. We obtained 270 (tumor = 160, normal = 110) WSIs of breast cancer from the Camelyon16 [18] dataset. We then extracted millions of small positive and negative image patches with a size of 224 × 224 pixels (40 × magnification) to pre-train the model for classifying tumor or normal tissues. The pre-trained model was fine-tuned using 100,000 image patches from 67 annotated WSIs from GDPH.

We used the OTSU method [19] to obtain the tissue region mask of WSIs. A window with a size of 224 × 224 pixels was slid without overlapping area on the whole tissue region. We used the trained model to predict the image patch under a sliding window, and the predicted probability was generated for each image patch. The predicted probability heatmap was further generated for each histopathological image. Finally, we binarized the predicted heatmap using the OTSU method, and retained the largest connected region as the tumor mask for each WSI. The framework is shown in Fig. 1a.

### Multi-scale texture feature extraction

The multi-scale texture feature extraction process is shown in Fig. 1b. Based on the results of tumor region segmentation, several image patches were acquired at magnifications of 2.5 × , 10 × , and 40 × . Color normalization [20] was performed for these patches to reduce the effect of staining differences on the texture distribution of images. In the case of 2.5 × magnification, the image of the whole tumor region was acquired directly. In the cases of 10 × and 40 × magnifications, we obtained image patches with a size of 1024 × 1024 pixels in the tumor region. To facilitate the acquisition of relatively dense image patches in the tumor region, the image patch with above 75% tissue area was used in this study. In the case of 40 × magnification, we randomly sampled 200 patches to reduce computational time for each WSI and avoid potential subjective bias [14].

We automatically extracted 68 texture features of tumor regions at each scale, including texture features such as first-order statistics (n = 17), gray level co-occurrence matrix (GLCM, n = 7), and gray level run length matrix (GLRLM, n = 44). First-order statistics features describe the distribution of pixel intensities within an image region. GLCM-based features consider the variation in pixel grey levels within a certain distance. GLRLM-based features quantify gray level runs defined as the number of consecutive pixels with the same gray level. Overall, a total of 204 texture features were extracted at three scales (i.e., 2.5 × , 10 × , and 40 × magnifications). Details of these features are provided in Additional file 1: Section 2.

### Feature selection and signature construction

To regularize the number of features proportionate to sample size, the features related to prognosis were selected through the least absolute shrinkage and selection operator (LASSO) method with tenfold cross-validation from the discovery set (Fig. 1c). Before feature selection, we normalized the feature values based on the Z-score method. Furthermore, it was crucial to visualize texture features related to prognosis so that all the clinicians could understand them. We quantified and visualized the selected texture features by the violin plot and feature heatmap.

MPIS was computed via a weighted linear combination of the discriminative texture features and their corresponding coefficients. The median value of MPIS in the discovery set was determined as the cut-off for distinguishing patients by risk level, with any value greater than the cut-off categorized as high-risk and any value equal to or less than the cut-off categorized as low-risk. The threshold identified from the discovery set was then applied to the external validation sets to distinguish high-risk and low-risk groups.

### Histo-genomic analysis

For the TCGA cohort, there were 244 patients available with normalized messenger ribonucleic acid (mRNA) expression data, after matching with the amount of TCGA data in survival analysis. We removed the genes whose mRNA expression levels were 0 in patient samples to explore the associations between gene expression of biological pathways and texture features derived from the histopathological image. First, patients were categorized as high-risk or low-risk according to the MPIS. We used the Wilcoxon rank-sum test to identify genes that were significantly differentially expressed across the high-risk and low-risk groups. The Benjamini & Hochberg method was employed to adjust *P*-value. We then used the differentially expressed genes (DEGs) for Gene Ontology (GO) enrichment analysis [21] to identify the biological pathways with over-represented genes in the gene set. Based on the identified pathways, we selected the ones potentially representative of biological processes related to tumor growth and development. Finally, we assessed the associations between the gene expression of biological pathways and the image-derived texture features by single-sample gene set enrichment analysis (ssGSEA) [22]. A ssGSEA enrichment score within each gene set was calculated for each patient, which assessed the degree to which member gene of a gene set in a sample was coordinately upregulated or downregulated. We used the Wilcoxon rank-sum test to select the significant differentially expressed pathways related to the image-derived texture features.

### Statistical analysis

Categorical data were reported as count (percentage). Differences in age, sex, smoking status, tumor site, treatment, differentiation, and TNM stage between four cohorts were evaluated through Pearson's chi-squared test or Fisher’s exact test, where appropriate. The data distribution of MPIS corresponding to different tumor differentiation degrees was also analyzed by the independent samples t-test. We used the log-rank test to estimate differences in OS between the high-risk and low-risk groups for Kaplan–Meier survival analysis. The prognostic abilities of MPIS and other clinical variables (i.e., age, sex, smoking status, tumor site, treatment, differentiation, and TNM stage) were assessed via univariable analysis. Then, the factors with *P* < 0.05 in the univariable analysis were adopted in the multivariable analysis. Akaike information criterion (AIC) was used in multivariable analysis to determine and evaluate the independent prognostic factors.

In the discovery set, a full model was established by incorporating the independent factors selected in the multivariable analysis, and the clinical model was built by independent clinicopathological variables. The full model and the clinical model were validated in the three independent external validation sets. Harrell’s concordance index (C-index) was used to determine the discriminative ability of models. The prognostic accuracy was evaluated using the time-dependent receiver operating characteristic (ROC) curve and area under the curve (AUC) at 5-year OS.

We conducted statistical analysis using R software (version 4.1.2, http://www.R-project.org) [23]. The packages of R software used for statistical analysis included glmnet, cutoff, survival, survminer, rms, timeROC, and vioplot. A factor was reported as statistically significant when the two-sided *P* < 0.05.

## Results

### Patients

We summarized the qualified patients in this study after applying all inclusion and exclusion criteria. The process is shown in (Additional file 1: Figure S1). The discovery set (n = 111) was established from GDPH, and employed for feature discovery and model training. Three independent cohorts were used for validating the trained model, collected from YNCH, SXCH, and TCGA. The three cohorts are denoted as external validation set V_1_ (n = 115), external validation set V_2_ (n = 116), and external validation set V_3_ (n = 246). Table 1 shows the detailed distributions of demographic and clinicopathological variables in the four cohorts. Significant differences were observed among the four cohorts in all included clinical characteristics, except for sex (*P* = 0.1603) and tumor site (*P* = 0.2230).

**Table 1:** Distributions of demographic and clinicopathological variables of patients with resectable LUAD in the discovery set and the three external validation sets

Variable	Discovery set N (%)	Validation set V_1_ N (%)	Validation set V_2_ N (%)	Validation set V_3_ N (%)	P
Number of patients	31/111*	30/115*	37/116*	80/246*	
Age					< **0.0001**^**#**^
< 65 years	41(36.9)	19(16.5)	78(67.2)	124(50.4)	
≥ 65 years	70(63.1)	96(83.5)	38(32.8)	122(49.6)	
Sex					0.1603^#^
Male	58(52.3)	58(50.4)	66(56.9)	110(44.7)	
Female	53(47.7)	57(49.6)	50(43.1)	136(55.3)	
Smoking Status					< **0.0001**^**†**^
Ever	32(28.8)	41(35.7)	56(48.3)	213(86.6)	
Never	79(71.2)	74(64.3)	60(51.7)	30(12.2)	
Unknown	0(0.0)	0(0.0)	0(0.0)	3(1.2)	
Tumor site					0.2230^**†**^
Upper/Middle	72(64.9)	65(56.5)	64(55.2)	160(65.0)	
Lower	39(35.1)	50(43.5)	52(44.8)	84(34.2)	
Unknown	0(0.0)	0(0.0)	0(0.0)	2(0.8)	
Treatment					< **0.0001**^**†**^
Surg. Only	76(68.5)	56(48.7)	55(47.4)	139(56.5)	
Surg. + Chemo	35(31.5)	59(51.3)	61(52.6)	87(35.4)	
Unknown	0(0.0)	0(0.0)	0(0.0)	20(8.1)	
Differentiation					< **0.0001**^**#**^
G1/G2	86(77.5)	60(52.2)	70(60.3)	NA	
G3/G4	22(19.8)	40(34.8)	46(39.7)	NA	
Unknown	3(2.7)	15(13.0)	0(0.0)	NA	
TNM stage					< **0.0001**^**#**^
I	79(71.2)	70(60.9)	49(42.2)	145(58.9)	
II	15(13.5)	17(14.8)	38(32.8)	65(26.4)	
III	17(15.3)	28(24.3)	29(25.0)	36(14.6)	

Bold value represents the significant difference is observed among the four cohorts in the corresponding clinical characteristic

*Surg.* + *Chemo* surgery and chemotherapy, *G1/G2* well-moderately differentiated, *G3/G4* poorly undifferentiated, *NA* not applicable

^*^Number of endpoint events (i.e., OS) that occurred/total number of patients

^**#**^The *P*-value is obtained using Pearson’s chi-squared test.

^**†**^The *P*-value is obtained using Fisher’s exact test

### Feature selection and signature construction

A set of eight potential predictors was selected from 204 multi-scale texture features using the LASSO method, namely glrlm_SRLGLE_90_2.5, glrlm_SRLGLE_90_40, glcm_dissimilarity_0_2.5, Kurtosis_10, glrlm_LRHGLE_90_2.5, glrlm_SRE_0_40, glcm_ASM_0_2.5, and Percentile_10th_40 (see in Additional file 1: Table S1 for specific definitions of these texture features). These texture features and corresponding regression coefficients are shown in (Additional file 1: Table S2). MPIS was computed for each patient through a linear combination of these feature values, weighted by the corresponding regression coefficients. The median value (-0.061) of MPIS in the discovery set was taken as the cut-off for stratifying patients.

**Fig. 2:**
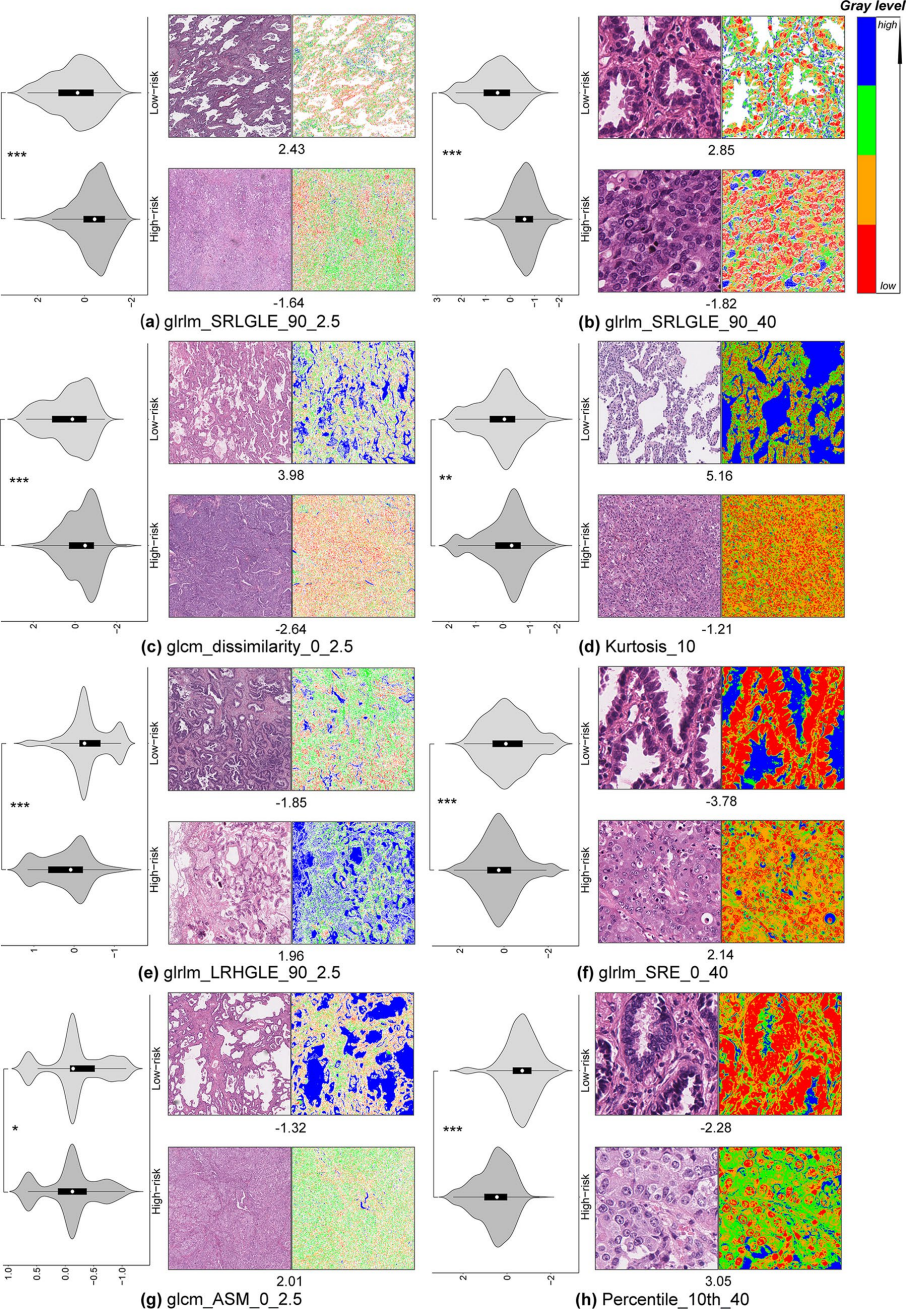
Graphical overview of the eight image texture features: **a** glrlm_SRLGLE_90_2.5, **b** glrlm_SRLGLE_90_40, **c** glcm_dissimilarity_0_2.5, **d** Kurtosis_10, **e** glrlm_LRHGLE_90_2.5, **f** glrlm_SRE_0_40, **g** glcm_ASM_0_2.5, **h** Percentile_10th_40. For each texture feature, a violin plot shows the distribution of the feature quantified in all patient samples. Furthermore, tissue image patches corresponding to high and low values and the correlated texture feature map are shown. Two tissue image patches are taken from high-risk and low-risk patients. The color coding (i.e., red, orange, green, and blue) of the feature map corresponds to the gray level (from low to high) of the image. The *P*-value is computed by the independent samples t-test. A significant difference in feature distribution between the high-risk and low-risk groups is observed for each texture feature. ***Represents *P* < 0.001, **represents *P* < 0.01, and *represents *P* < 0.05

As shown in Fig. 2, we quantified and visualized the image texture features in which significant differences were observed between the two representative images from the high-risk and low-risk groups determined by the corresponding feature. The low-risk example had higher feature values than the high-risk example in the cases of features glrlm_SRLGLE_90_2.5, glrlm_SRLGLE_90_40, glcm_dissimilarity_0_2.5, and Kurtosis_10 (Fig. 2(a–d)), while had lower feature values than the high-risk example in the cases of features glrlm_LRHGLE_90_2.5, glrlm_SRE_0_40, glcm_ASM_0_2.5, Percentile_10th_40 (Fig. 2(e–h)).

### Evaluation and validation of MPIS

**Fig. 3:**
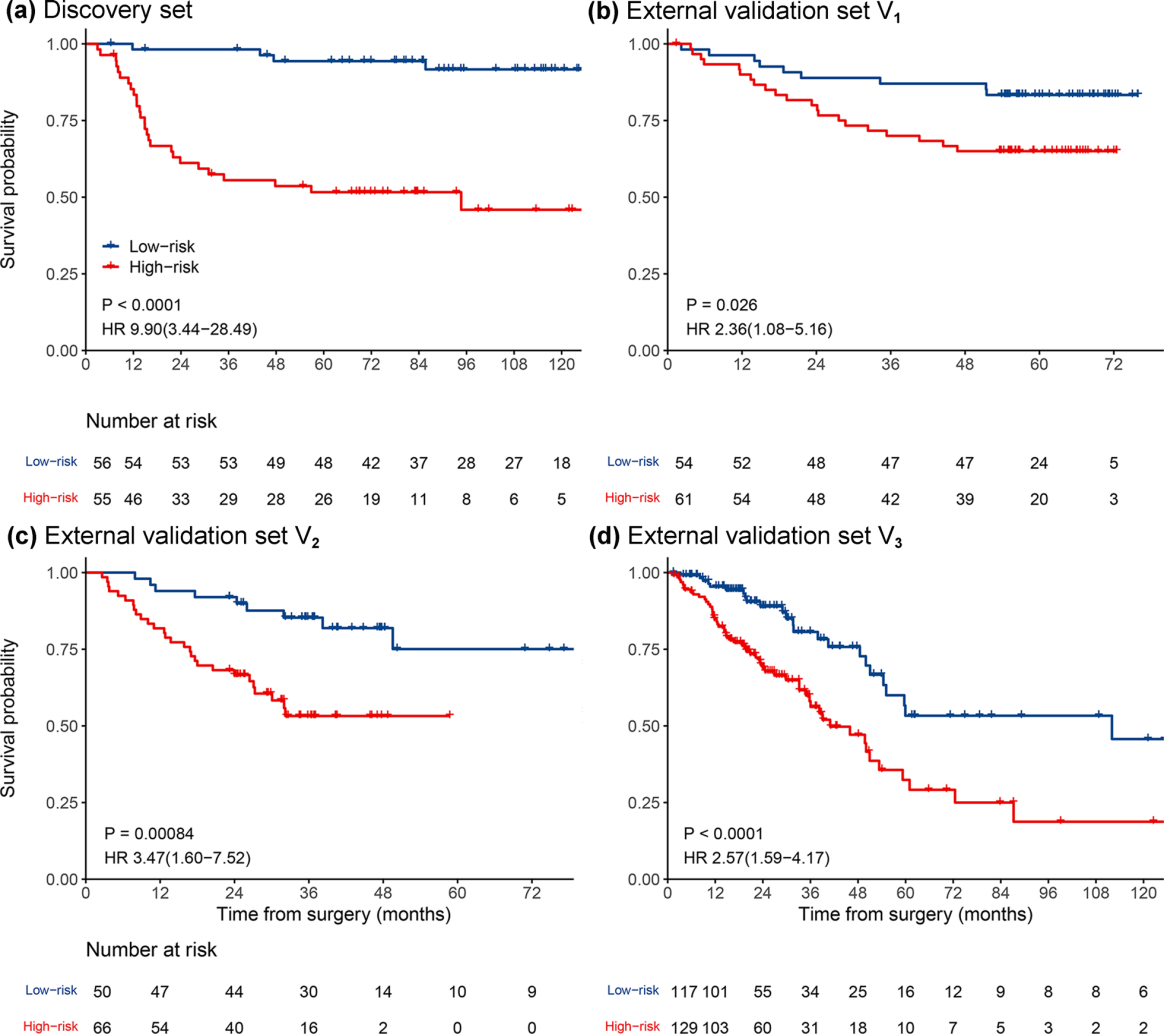
Kaplan–Meier curves of patients stratified by MPIS in the **a** discovery set, **b** external validation set V_1_, **c** external validation set V_2_, and **d** external validation set V_3_

Kaplan–Meier curves for predicting OS by MPIS showed that the low-risk group had a significantly better survival rate compared with the high-risk group (Fig. 3). On univariable analysis, MPIS was statistically significant in the four cohorts, as shown in Table 2. MPIS was associated with OS in the discovery set (hazard ratio [HR], 9.90; 95% confidence interval [CI], 3.44–28.49; *P* < 0.0001). Furthermore, MPIS was also prognostic of OS on the external validation set V_1_ (HR, 2.36; 95%CI, 1.08–5.16; *P* = 0.0312), external validation set V_2_ (HR, 3.47; 95%CI, 1.60–7.52; *P* = 0.0016), and external validation set V_3_ (HR, 2.57; 95%CI 1.59–4.17; *P* = 0.0001). Multivariable analysis was conducted using factors (treatment, TNM stage, differentiation, MPIS) that achieved statistical significance (*P* < 0.05) in univariable analysis. On multivariable analysis, we further demonstrated that MPIS was an independent prognostic factor on the discovery set (HR, 5.32; 95% CI 1.17–16.44; *P* = 0.0037), external validation set V_1_ (HR, 2.63; 95% CI 1.10–6.29; *P* = 0.0292), external validation set V_2_ (HR, 2.99; 95% CI 1.34–6.66; *P* = 0.0075), and external validation set V_3_ (HR, 1.93; 95% CI 1.15–3.23; *P* = 0.0125).

**Table 2:** Univariable and multivariable analysis for OS on the discovery set and the three external validation sets

	Discovery set		Validation set V_1_		Validation set V_2_		Validation set V_3_	
	HR (95%CI)	P-value	HR (95%CI)	P-value	HR (95%CI)	P-value	HR (95%CI)	P-value
Univariable Analysis								
Age								
< 65 years vs. ≥ 65 years	1.54(0.76–3.11)	0.2309	1.20(0.49–2.93)	0.6910	1.20(0.61–2.37)	0.5907	1.17(0.76–1.83)	0.4753
Sex								
Male vs. Female	0.62(0.30–1.28)	0.1962	0.95(0.46–1.95)	0.8944	0.40(0.20–0.84)	0.0149	0.69(0.45–1.08)	0.1041
Smoking status								
Never vs. Ever	1.51(0.72–3.15)	0.2730	0.69(0.32–1.51)	0.3514	2.15(1.11–4.20)	0.0241	1.77(0.77–4.08)	0.1814
Tumor site								
Upper/Middle vs. Lower	0.83(0.39–1.76)	0.6205	0.99(0.48–2.05)	0.9865	0.70(0.35–1.37)	0.2940	1.30(0.83–2.04)	0.2474
Treatment								
Surg. vs. Surg. + Chemo	4.46 (2.16–9.24)	< 0.0001	1.78(0.85–3.75)	0.1268	1.47(0.76–2.87)	0.2539	1.92(1.19–3.08)	0.0074
Differentiation								
G1/G2 vs. G3/G4	7.00(3.40–14.40)	< 0.0001	1.60(0.74–3.46)	0.2296	3.07(1.58–5.97)	0.0010	NA	
TNM stage								
II vs. I	4.93(1.90–12.81)	0.0011	2.67(0.97–7.35)	0.0572	1.20(0.53–2.72)	0.6669	2.88(1.75–4.72)	< 0.0001
III vs. I	9.82(4.33–22.28)	< 0.0001	4.55(2.02–10.26)	0.0003	2.51(1.16–5.47)	0.0201	3.54(1.95–6.41)	< 0.0001
MPIS								
Low vs. High	9.90(3.44–28.49)	< 0.0001	2.36(1.08–5.16)	0.0312	3.47(1.60–7.52)	0.0016	2.57(1.59–4.17)	0.0001
Multivariable analysis								
Differentiation								
G1/G2 vs. G3/G4	2.47(1.12–5.49)	0.0259			3.49(1.72–7.06)	0.0005	NA	
TNM stage								
II vs. I	2.72(0.99–7.44)	0.0521	2.60(0.87–7.78)	0.0871	1.25(0.54–2.87)	0.6031	2.20 (1.30–3.74)	0.0035
III vs. I	3.12(1.23–7.92)	0.0166	5.30(2.22–12.66)	0.0002	3.24(1.45–7.28)	0.0043	3.08 (1.68–5.64)	0.0003
MPIS								
Low vs. High	5.32(1.72–16.44)	0.0037	2.63(1.10–6.29)	0.0292	2.99(1.34–6.66)	0.0075	1.93 (1.15–3.23)	0.0125

*HR* hazard ratio, *CI* confidence interval, *Surg.* + *Chemo* surgery and chemotherapy, *G1/G2* well-moderately differentiated, *G3/G4* poorly undifferentiated, *NA* not applicable

MPIS could predict OS in patients with TNM stage I and early-stage (TNM stages I and II) LUAD (Additional file 1: Figures S2, S3). For early-stage LUAD patients, the survival outcomes of patients in the high-risk group were significantly worse than those in the low-risk group. Although no statistical association was found between MPIS and OS in the external validation set V_1_ (*P* = 0.13), we could still observe a clear trend for poor prognosis in the high-risk group. For TNM stage I LUAD patients, the low-risk group had a better prognosis. Additionally, when stratifying patients by clinicopathological variables, including age (≥ 65 or < 65 years), sex (female or male), smoking status (ever smoke or never smoke), treatment (surgery alone or received chemotherapy), and differentiation (well-moderately differentiated or poorly undifferentiated), MPIS was associated with OS in most of the subgroups (Additional file 1: Figures S4–S8).

**Fig. 4:**
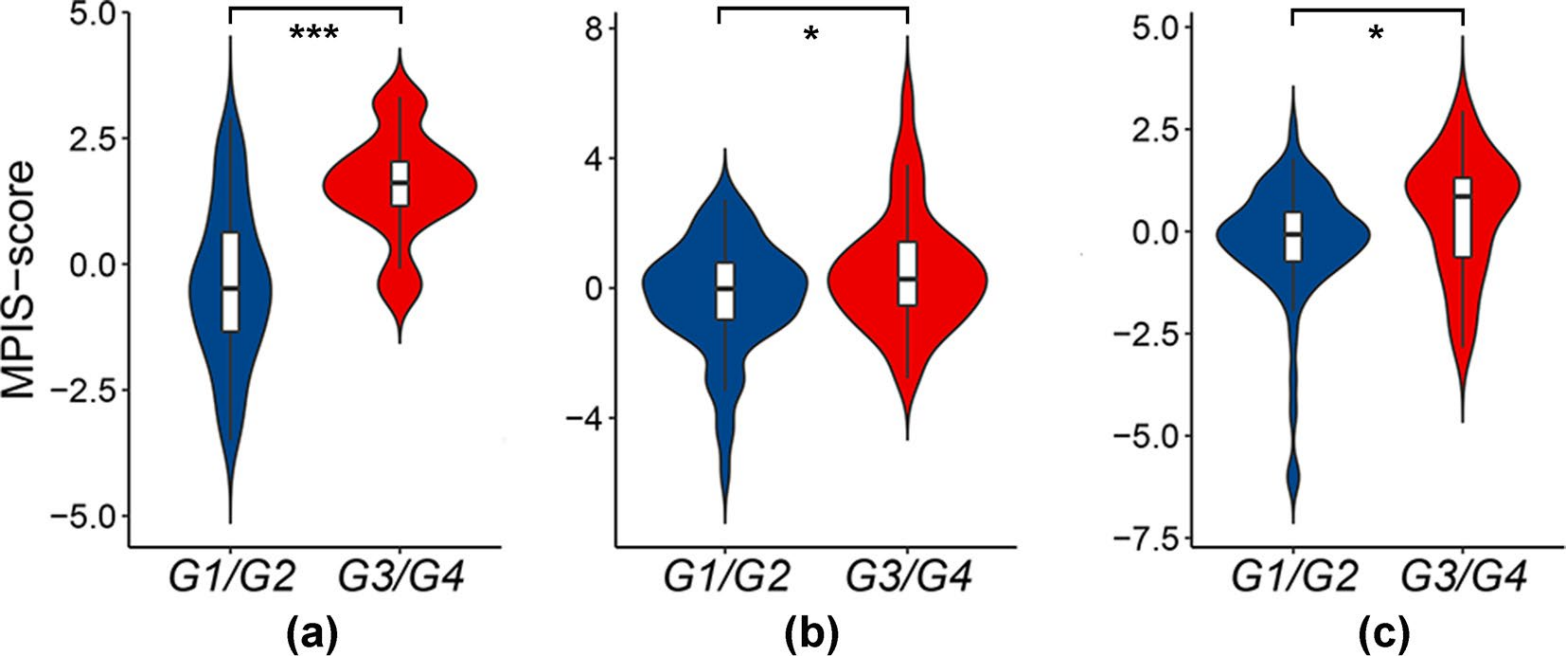
MPIS distribution across the well-moderately differentiated and poorly undifferentiated LUAD patients. The distribution of MPIS is significantly different between the well-moderately differentiated and poorly-undifferentiated groups on the **a** discovery set (*P* < 0.0001), **b** external validation set V_1_ (*P* = 0.0314), and **c** external validation set V_2_ (*P* = 0.0104). The *P*-value is obtained using the independent samples t-test. G1/G2, well-moderately differentiated; G3/G4, poorly undifferentiated

In addition, MPIS was significantly higher in the poorly undifferentiated group compared with the well-moderately differentiated groups on the discovery set (*t* = −7.02; *P* < 0.0001), external validation set V_1_ (*t* = −2.19; *P* = 0.0314), and external validation set V_2_ (*t* = −2.61; *P* = 0.0104). The violin plots in Fig. 4 show the distribution of MPIS across the well-moderately differentiated and poorly undifferentiated LUAD patient groups.

### Evaluation and validation of the full model

**Fig. 5:**
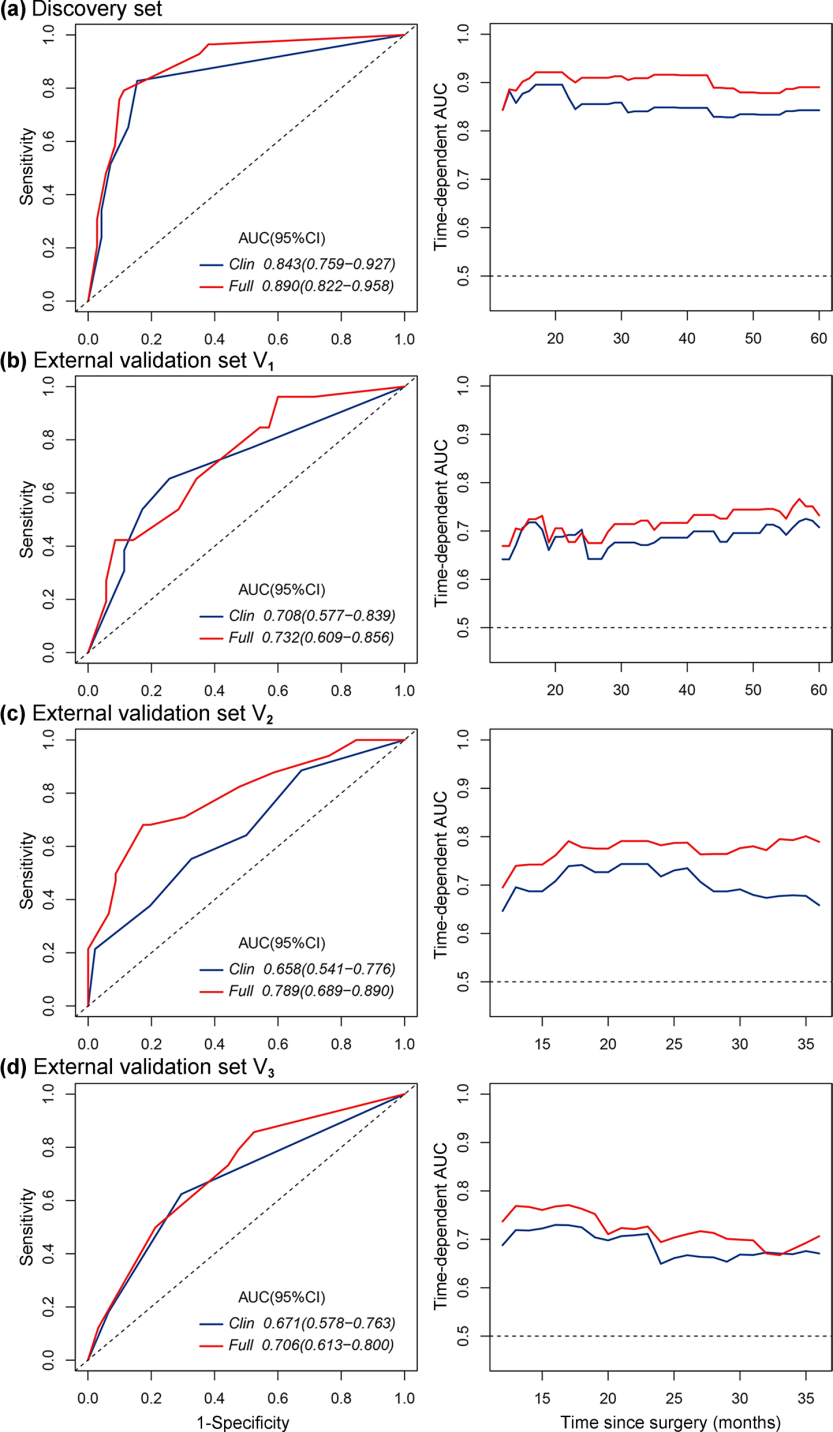
Time-dependent ROC curves and AUC curves of models in the **a** discovery set, **b** external validation set V_1_, **c** external validation set V_2_, and **d** external validation set V_3_. Time-dependent ROC curves are evaluated for 5-year OS (or for 3-year OS), and time-dependent AUC curves are plotted for 12–60 months (or for 12–36 months)

Using stepwise regression based on the AIC, independent prognostic factors were identified, including MPIS, differentiation, and TNM stage (Table 2). In the discovery set, we built the full model incorporating the above independent factors, and established the clinical model incorporating two clinicopathological variables (i.e., differentiation and TNM stage). It was observed that the C-index of the full model (0.837; 95% CI 0.784–0.890; Table 3) was higher than that of the clinical model (C-index, 0.798; 95% CI 0.729–0.867), and the AIC of the full model was smaller than that of the clinical model (235.991 vs. 244.905; Table 3). Therefore, the full model showed higher discrimination and calibration than the clinical model. Meanwhile, we demonstrated that integrating the MPIS into the clinical model significantly improved the prediction for OS (*P* = 0.0010, likelihood ratio test), as shown in Table 3. Time-dependent ROC curves at 60 months and time-dependent AUC curves at different times were plotted, as shown in Fig. 5a. The full model (AUC, 0.890; 95%CI, 0.822–0.958; for 5-year OS) showed significantly improved predictive performance compared with the clinical model (AUC, 0.843; 95%CI, 0.759–0.927; for 5-year OS). Furthermore, we visualized the full model and the clinical model as nomograms to facilitate the application of the full model (Additional file 1: Figure S9).

**Table 3:** Performance of models in the discovery set and the three external validation sets

		Clinical model	Full model
*Discovery set*	C-index(95%CI)	0.798(0.729–0.867)	0.837(0.784–0.890)
	AIC	244.905	235.991
	*P-value*	0.0010	
*External validation set V*_*1*_	C-index(95%CI)	0.679(0.573–0.784)	0.704(0.608–0.801)
	AIC	222.908	219.568
	*P-value*	< 0.0001	
*External validation set V*_*2*_	C-index(95%CI)	0.666(0.572–0.761)	0.728(0.646–0.811)
	AIC	313.815	307.537
	*P-value*	< 0.0001	
*External validation set V*_*3*_	C-index(95%CI)	0.669(0.606–0.731)	0.696(0.632–0.760)
	AIC	722.453	717.869
	*P-value*	< 0.0001	

Clinical model: TNM stage + differentiation; Full model: TNM stage + differentiation + MPIS. Note that information related to tumor differentiation was unavailable in the external validation set V_3_. The *P*-value is calculated to compare the difference between the clinical model and the full model by the likelihood ratio test

*CI* confidence interval, *AIC* Akaike information criterion

We further validated the performance of the full model in the independent external validation sets (Table 3). The full model had better discriminative and calibration (V_1_: C-index, 0.704 vs. 0.679; *P* < 0.0001, likelihood ratio; AIC, 219.568 vs. 222.908; V_2_: 0.728 vs. 0.666; *P* < 0.0001; 307.537 vs. 313.815) than the clinical model in the two external validation sets. In Figs. 5b, c, AUC curves showed that the full model had better performance at every time point in the two external validation sets (V_1_: AUC, 0.732 vs. 0.708; for 5-year OS; V_2_: 0.789 vs. 0.658; for 3-year OS). Besides, due to the lack of information related to tumor differentiation in the external validation set V_3_, the full model was established with two variables (i.e., TNM stage and MPIS), and the clinical model was established with one variable (i.e., TNM stage). It can be observed that the full model (C-index, 0.696 vs. 0.669; AIC, 717.869 vs. 722.453; likelihood ratio, *P* < 0.0001; AUC, 0.706 vs. 0.671; for 3-year OS) still outperformed the clinical model in terms of discrimination and calibration (Table 3, Fig. 5d).

To further demonstrate the incremental value of MPIS, we also selected features from the individual scale, calculated the corresponding single-scale pathology image signature, and constructed single-scale models including a 2.5 × model, a 10 × model, and a 40 × model. The feature selected at each scale and their corresponding coefficients are detailed in (Additional file 1: Tables S3–S5 ). The single-scale texture signature at 2.5 × , 10 × , and 40 × magnifications were associated with the OS in the discovery set and the three external validation sets (Additional file 1: Figures S10–S12). Compared to the single-scale models, the full model still had a higher AUC value at most time points (Additional file 1: Figure S13).

### Histo-genomic analysis

**Fig. 6:**
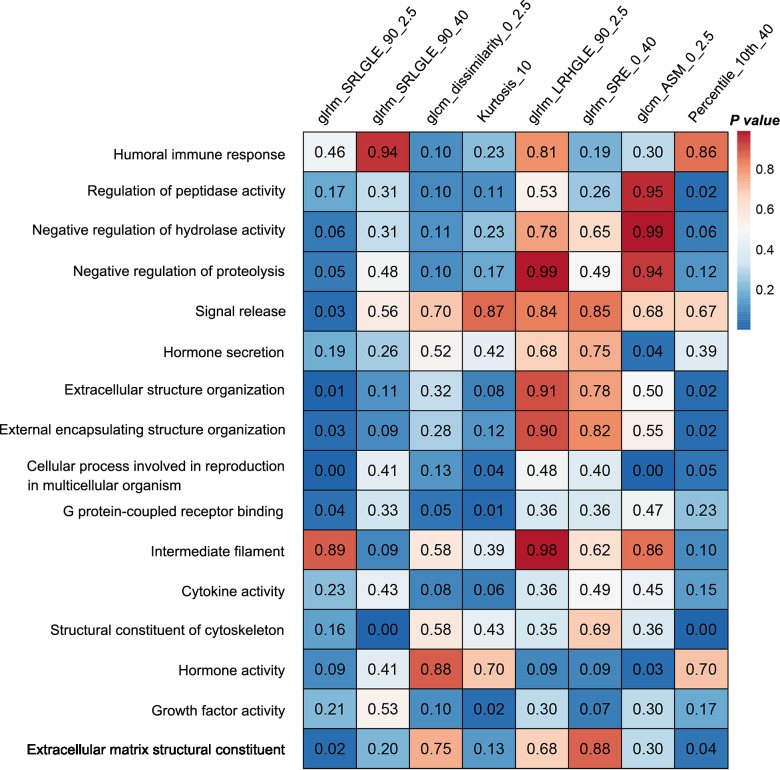
Associations between biological pathways and the identified texture features. The strengths of associations of biological pathways (shown in rows) with texture features (shown in columns) were shown by ssGSEA analysis. Wilcoxon rank-sum test *P*-values are shown, where *P* < 0.05 indicates an association exists between the texture feature of the tumor region derived from histopathological images and the biological pathway

The transcriptomic data consisted of 19,645 annotated genes across TCGA-LUAD. We performed differential gene expression analysis, and found 194 DEGs between the MPIS-defined high-risk and low-risk groups. These DEGs identified 16 significant biological pathways through GO enrichment analysis. These significant pathways were involved in cytokine activity, cell proliferation, metabolism, growth, division, and extracellular matrix structure, and they were considered to be correlated with the growth and development of tumors. Specifically, DEGs showed significant enrichment in biological pathways such as humoral immune response, regulation of peptidase activity, signal release, and extracellular structure organization (Additional file 1: Figure S14). The full list of DEGs and pathways is presented in Additional file 2. Furthermore, we evaluated the associations between the gene expression of biological pathways and the image-derived texture features with ssGSEA. We used 16 biological pathways to calculate the enrichment scores for each of the eight texture features used to construct the MPIS. As shown in Fig. 6, the texture features of the tumor region derived from histopathological images (i.e., glrlm_SRLGLE_90_2.5, glcm_ASM_0_2.5, and Percentile_10th_40) were significantly associated with biological pathways such as extracellular structure organization, structural constituent of cytoskeleton, hormone activity, and extracellular matrix structural constituent.

## Discussion

Accurate prognosis for resectable LUAD could guide clinical decision-making and improve risk stratification. Although morphological examination of tumors in routine histopathological slides by pathologists could help predict cancer behavior, manual review fails to quantify sub-visual features of tumors. In this study, we developed a fully automated pipeline to analyze the tumor and its microenvironment through extracting multi-scale texture information from the tumor region in H&E-stained WSIs. We used the texture information to construct MPIS and evaluated its prognostic ability for predicting OS in patients with resectable LUAD. The results demonstrated that MPIS was an independent prognostic factor for OS. Moreover, integrating MPIS with clinicopathological variables improved the prognostic stratification in resectable LUAD. In addition, the image-derived multi-scale texture features were associated with biological pathways affecting tumor development. We validated the prognostic model in four independent cohorts, including large multi-institutional data from the TCGA cohort. MPIS was an independent prognostic factor in all four cohorts, even though there were statistically significant differences among these four cohorts (Table 1). At the same time, we observed significant stratification in most subgroups (Additional file 1: Figure S2–S8). This suggested that MPIS is a robust prognostic biomarker of OS in resectable LUAD and can be easily generalized to other centers.

In recent years, many histopathological biomarkers have been developed for the prognosis of patients with lung cancer. For instance, Yu et al. [13] and Chen et al. [24] employed CellProfiler [25–27] software to quantitatively measure cellular phenotypes in histopathological images, and correlated these features with prognosis. Several studies [28–30] captured cellular-level feature descriptors from segmented nuclei for predicting prognosis in early-stage non-small cell lung cancer. In addition, Wang et al. [31] have provided insights into the relationship between tumor shape and prognosis in patients with LUAD. However, most of these potential biomarkers are mainly focused on single-scale information, on either the cellular level or the tissue level of histopathological images. Differently, this study leveraged multi-scale texture features from tumor regions to construct an image signature for prognosticating OS of LUAD patients. The motivation for quantifying multi-scale texture features was based on routine examination of histopathological slides by pathologists. Pathologists generally first observe the whole slide tissue at the tissue level with low magnification, and then selectively examine the morphological features at the cellular level with high magnification. Specifically, a 2.5 × magnification image contains global information about the whole tumor, a 10 × magnification image contains the characteristics of the tumor region at the tissue level, and a 40 × magnification image contains tumor features at the cellular level. Compared with single-scale texture signatures, we found that MPIS could improve the prognostic stratification in resectable LUAD, and the full model that integrated MPIS and clinicopathological variables had better prediction power (Additional file 1: Figure S13). This seems to indicate that MPIS can effectively capture multi-scale information from the cellular level to the tissue level in histopathological images, and can comprehensively assess morphological characterization of tumors.

Over the past few years, different deep learning approaches have been proposed to quantify tumors and their surrounding microenvironment, resulting in various potential biomarkers based on deep features for prognosis [32–34]. For example, Coudray et al. [15] demonstrated that deep learning models could assist pathologists in automatically detecting cancer subtypes or gene mutations. Shi et al. [33] proposed an efficient and labor-saving deep learning method for providing a valuable means of patient risk stratification. Nevertheless, they only enabled subjectively provide hypothetical explanations based on slide-by-slide qualitative assessments, let alone objectively connect deep features to biological phenomena, although class activation maps [35, 36] could visualize interested image regions in end-to-end CNN models.

In contrast, our work could directly correlate with biological concepts, and provide interpretability in histopathology and genomics. On the one hand, we tracked down observable texture features from a histopathological standpoint to reduce the risk of spurious correlations. Specifically, we observed significant differences in the distribution of MPIS between the well-moderately differentiated and poorly undifferentiated groups (Fig. 4). This seems to suggest that a significant association exists between MPIS and tumor differentiation performed by pathologists. For example, the abundance and spatial distribution of tumor cells and the growth pattern of stroma might be reflected in the texture features of WSIs. MPIS could discriminate the degree of tumor differentiation by quantifying these texture features. Furthermore, we found that the selected multi-scale texture features might be directly correlated with biological phenomena by quantifying phenotypic information in histopathological images, and could provide interpretability for investigators. More specifically, the feature glrlm_ SRLGLE measured the pattern of consecutive pixels with lower gray value in an image. In the context of histopathological images, a larger glrlm_SRLGLE feature value might reflect the sparser distribution of cells in the tissue image. This biological phenomenon might indicate a lepidic or acinar growth pattern of LUAD (Fig. 2a, b). The feature glcm_ASM measured the gray scale uniformity of the image. A larger value indicated a higher degree of uniformity. As shown in Fig. 2g, the bottom figure had a higher glcm_ASM feature value. One may observe that the tissues and cells grow relatively more densely in the tumor compared with that of the top figure, and the tumor growth pattern seems to be solid.

On the other hand, we also investigated the biological pathways that might drive tumor development by histo-genomics analysis, which further elaborated the interpretability of texture features from a genomics perspective. In this study, the selected texture features were associated with significant biological pathways affecting tumor development. For instance, the extracellular matrix structural constituent was significantly associated with the features glrlm_SRLGLE_90_2.5, and Percentile_10th_40. Gene expression of these pathways has been shown to affect tumors and their microenvironment [37], possibly suggesting that the stromal tissue structure influences the texture distribution of the tumor region. Moreover, the cellular microenvironment constantly regulates cell growth, apoptosis, and differentiation by cytoskeletal remodeling [38]. We found a significant correlation between the structural constituent of cytoskeleton and the image-derived texture features such as glrlm_SRLGLE_90_40, clearly suggesting that the texture feature might be driven by pathways related to cellular apoptosis and differentiation. Cytokine activity [39, 40], which could be another latent reason for affecting the texture distribution of the tumor region, reflects the survival, growth, differentiation, and effector function of tissues and cells.

This study had some limitations. First, our study was based on retrospective cohorts, which may be impressionable to bias from some risk variables and the loss of follow-up. In the future, we will further validate our model in larger cohorts or a prospective study. Second, MPIS was developed and validated with data from different institutions, which meant some relevant demographic parameters were unavailable in some datasets. Third, this study employed a deep learning method based on transfer learning to segment the tumor region. However, pathologists still needed to annotate a small number of slides to fine-tune the segmentation model, improving the model's performance. In the future, we will use weakly supervised or unsupervised learning models for quantitative analysis to minimize the labeling work of pathologists.

## Conclusions

In summary, we developed and validated MPIS, which could successfully stratify patients with resectable LUAD into high-risk and low-risk groups with significant differences in OS. MPIS was an independent prognostic factor for OS, and the integration of MPIS with clinicopathological variables improved the prognostic stratification for patients with resectable LUAD. The study demonstrated that MPIS was a comprehensive, robust, and interpretable predictor and could contribute to the field of precision oncology by helping to improve the quality of individualized postoperative care.

## Supplementary Information

**Additional file 1: Section 1.** Inclusion and exclusion criteria. **Section 2.** Texture feature definition. **Figure S1.** Data preparation and demographics of all cohorts. **Figure S2.** Kaplan–Meier curves of patients stratified by MPIS in the subgroup of patients with TNM stage I LUAD in the (a) discovery set; (b) external validation set V_1_; (c) external validation set V_2_; (d) external validation set V_3_. **Figure S3.** Kaplan–Meier curves of patients stratified by MPIS in the subgroup of patients with early-stage LUAD in the (a) discovery set; (b) external validation set V_1_; (c) external validation set V_2_; (d) external validation set V_3_. **Figure S4.** Kaplan–Meier curves of patients stratified by MPIS in the subgroups: (a) patients with age < 65 years in the discovery set; (b) patients with age ≥ 65 years in the discovery set; (c) patients with age < 65 years in the external validation set V_1_; (d) patients with age ≥ 65 years in the external validation set V_1_; (e) patients with age < 65 years in the external validation set V_2_; (f) patients with age ≥ 65 years in the external validation set V_2_; (g) patients with age < 65 years in the external validation set V_3_; (h) patients with age ≥ 65 years in the external validation set V_3_. **Figure S5.** Kaplan–Meier curves of patients stratified by MPIS in the subgroups: (a) male sex in the discovery set; (b) female sex in the discovery set; (c) male sex in the external validation set V_1_; (d) female sex in the external validation set V_1_; (e) male sex in the external validation set V_2_; (f) female sex in the external validation set V_2_; (g) male sex in the external validation set V_3_; (h) female sex in the external validation set V_3_. **Figure S6.** Kaplan–Meier curves of patients stratified by MPIS in the subgroups: (a) non-smoker in the discovery set; (b) smoker in the discovery set; (c) non-smoker in the external validation set V_1_; (d) smoker in the external validation set V_1_; (e) non-smoker in the external validation set V_2_; (f) smoker in the external validation set V_2_; (g) non-smoker in the external validation set V_3_; (h) smoker in the external validation set V_3_. **Figure S7.** Kaplan–Meier curves of patients stratified by MPIS in the subgroups: (a) patients without adjuvant chemotherapy in the discovery set; (b) patients received adjuvant chemotherapy in the discovery set; (c) patients without adjuvant chemotherapy in the external validation set V_1_; (d) patients received adjuvant chemotherapy in the external validation set V_1_; (e) patients without adjuvant chemotherapy in the external validation set V_2_; (f) patients received adjuvant chemotherapy in the external validation set V_2_; (g) patients without adjuvant chemotherapy in the external validation set V_3_; (h) patients received adjuvant chemotherapy in the external validation set V_3_. **Figure S8.** Kaplan–Meier curves of patients stratified by MPIS in the subgroups: (a) patients with well-moderately differentiated cancer in the discovery set; (b) patients with poorly-undifferentiated cancer in the discovery set; (c) patients with well-moderately differentiated cancer in the external validation set V_1_; (d) patients with poorly-undifferentiated cancer in the external validation set V_1_; (e) patients with well-moderately differentiated cancer in the external validation set V_2_; (f) patients with poorly-undifferentiated cancer in the external validation set V_2_. **Figure S9.** The visualization of the full model (a) and clinical model (b) as nomograms for patients with resectable LUAD. **Figure S10.** Kaplan–Meier curves of patients stratified by single-scale pathology image signature at 2.5 × magnification in the (a) discovery set, (b) external validation set V_1_, (c) external validation set V_2_, and (d) external validation set V_3_. **Figure S11.** Kaplan–Meier curves of patients stratified by single-scale pathology image signature at 10 × magnification in the (a) discovery set, (b) external validation set V_1_, (c) external validation set V_2_, and (d) external validation set V_3_. **Figure S12.** Kaplan–Meier curves of patients stratified by single-scale pathology image signature at 40 × magnification in the (a) discovery set, (b) external validation set V_1_, (c) external validation set V_2_, and (d) external validation set V_3_. **Figure S13.** Time-dependent ROC curves and AUC curves of models in the (a) discovery set, (b) external validation set V_1_, (c) external validation set V_2_, and (d) external validation set V_3_. Time-dependent ROC curves are evaluated for 5-year OS (or for 3-year OS), and time-dependent AUC curves are plotted for 12 to 60 months (or 12 to 36 months). **Figure S14.** Significantly enriched biological pathways in Gene Ontology (GO) enrichment analysis. **Table S1.** The specific definitions of the selected texture features. **Table S2.** The LASSO Cox selected features and corresponding coefficients to construct the MPIS. **Table S3.** The LASSO Cox selected features and corresponding coefficients to construct the single-scale pathology image signature at 2.5 × magnification. **Table S4.** The LASSO Cox selected features and corresponding coefficients to construct the single-scale pathology image signature at 10 × magnification. **Table S5.** The LASSO Cox selected features and corresponding coefficients to construct the single-scale pathology image signature at 40 × magnification.

**Additional file 2: **The full list of DEGs and pathways.

## Data Availability

The histopathology images and clinical information of the TCGA cohort are available in a public repository from the Genomic Data Commons Data Portal (https://portal.gdc.cancer.gov/). All other data supporting the findings of this study are available from corresponding authors upon reasonable request. The source code for survival analysis can be accessed online: https://github.com/YuMeng-W/MPIS-LUNG.

## Abbreviations

LUAD: Lung adenocarcinoma
TNM: Tumor-node-metastasis
H&E: Hematoxylin and Eosin
WSI: Whole slide image
MPIS: Multi-scale pathology image texture signature
OS: Overall survival
GDPH: Guangdong Provincial People's Hospital
YNCH: Yunnan Cancer Hospital
SXCH: Shanxi Provincial Cancer Hospital
TCGA: The Cancer Genome Atlas
GLCM: Gray level co-occurrence matrix
GLRLM: Gray level run length matrix
LASSO: Least absolute shrinkage and selection operator
mRNA: Messenger ribonucleic acid
GO: Gene Ontology
DEG: Differentially expressed gene
ssGSEA: Single-sample gene set enrichment analysis
AIC: Akaike information criterion
C-index: Concordance index
ROC: Receiver operating characteristic curve
AUC: Area under the curve
