# Supplementary material for: Multi-scale pathology image texture signature is a prognostic factor for resectable lung adenocarcinoma: a multi-center, retrospective study
Source: J Transl Med. 2022 Dec 14;20:595. doi: 10.1186/s12967-022-03777-x (PMC9749333; doi:10.1186/s12967-022-03777-x)
Supplement: Supplementary file 1 — Additional file 1: Section 1. Inclusion and exclusion criteria. Section 2. Texture feature definition. Figure S1. Data preparation and demographics of all cohorts. Figure S2. Kaplan–Meier curves of patients stratified by MPIS in the subgroup of patients with TNM stage I LUAD in the (a) discovery set; (b) external validation set V1; (c) external validation set V2; (d) external validation set V3. Figure S3. Kaplan–Meier curves of patients stratified by MPIS in the subgroup of patients with early-stage LUAD in the (a) discovery set; (b) external validation set V1; (c) external validation set V2; (d) external validation set V3. Figure S4. Kaplan–Meier curves of patients stratified by MPIS in the subgroups: (a) patients with age < 65 years in the discovery set; (b) patients with age ≥ 65 years in the discovery set; (c) patients with age < 65 years in the external validation set V1; (d) patients with age ≥ 65 years in the external validation set V1; (e) patients with age < 65 years in the external validation set V2; (f) patients with age ≥ 65 years in the external validation set V2; (g) patients with age < 65 years in the external validation set V3; (h) patients with age ≥ 65 years in the external validation set V3. Figure S5. Kaplan–Meier curves of patients stratified by MPIS in the subgroups: (a) male sex in the discovery set; (b) female sex in the discovery set; (c) male sex in the external validation set V1; (d) female sex in the external validation set V1; (e) male sex in the external validation set V2; (f) female sex in the external validation set V2; (g) male sex in the external validation set V3; (h) female sex in the external validation set V3. Figure S6. Kaplan–Meier curves of patients stratified by MPIS in the subgroups: (a) non-smoker in the discovery set; (b) smoker in the discovery set; (c) non-smoker in the external validation set V1; (d) smoker in the external validation set V1; (e) non-smoker in the external validation set V2; (f) smoker in the external v [file 12967_2022_3777_MOESM1_ESM.pdf]

Supplementary Information for

Multi-scale pathology image texture signature is a prognostic factor for resectable lung adenocarcinoma: A multi-center, retrospective study

This file includes:

1. **Inclusion and exclusion criteria**
2. **Feature definition**
3. **Supplementary figures**
4. **Supplementary tables**

1. Inclusion and exclusion criteria

Inclusion criteria

(1) Patients who underwent surgery for lung adenocarcinoma with curative intent;
(2) Patients whose overall survival (OS) information was complete and available;
(3) AJCC TNM stage I–III;
(4) Follow-up registered;
(5) Age $\geq$ 18 years.

Exclusion criteria

(1) Patients with a history of previous pulmonary surgery, preoperative neoadjuvant therapy, or other malignancies;
(2) Surgical margins with residual tumor;
(3) Patients whose OS was less than 1 month;
(4) Missing hematoxylin and eosin (H&E)-stained whole-slide images (WSIs);
(5) The quality control of H&E-stained WSIs: excluding blurry, artifacts, poor staining, and insufficient tumor tissue.

2. Feature definition

Group 1: First-order statistics

First-order statistics included minimum, maximum, mean, median, range, 10th percentile, 90th percentile, interquartile range, energy, entropy, uniformity, skewness, kurtosis, variance, standard deviation, root mean squared (RMS), mean absolute deviation (MAD).

Group 2: Grey level co-occurrence matrix (GLCM)

Contrast

$$Contrast = \sum_i \sum_j (i-j)^2 P(i,j)$$

Dissimilarity

$$Dissimilarity = \sum_{i,j} |i-j| P(i,j)$$

Homogeneity

$$Homogeneity = \sum_{i,j} \frac{P(i,j)}{1+|i-j|}$$

Energy

$$Energy = \sqrt{\sum_{i,j} P(i,j)^2}$$

Correlation

$$Correlation = \sum_{i,j} \frac{(i-\mu i)(j-\mu j)P(i,j)}{\sigma_i \sigma_j}$$

Angular Second Moment (ASM)

$$ASM = \sum_{i,j} P(i,j)^2$$

Standard Deviation

$$Std = std(P(i,j))$$

where $P(i,j)$ represented the gray level co-occurrence matrix.

Group 3: Gray level run length matrix (GLRLM)

Short Run Emphasis (SRE)

$$SRE = \frac{\sum_{i,j} \frac{P(i,j|\theta)}{j^2}}{\sum_{i,j} P(i,j|\theta)}$$

Long Run Emphasis (LRE)

$$LRE = \frac{\sum_{i,j} j^2 P(i,j|\theta)}{\sum_{i,j} P(i,j|\theta)}$$

Gray Level Non-Uniformity (GLN)

$$GLN = \frac{\sum_i [\sum_j P(i,j|\theta)]^2}{\sum_{i,j} P(i,j|\theta)}$$

Run Level Non-Uniformity (RLN)

$$RLN = \frac{\sum_j [\sum_i P(i,j|\theta)]^2}{\sum_{i,j} P(i,j|\theta)}$$

Run Percentage (RP)

$$RP = \sum_{i,j} \frac{P(i,j|\theta)}{N_p}$$

Low Gray Level Run Emphasis (LGLRE)

$$LGLRE = \frac{\sum_{i,j} \frac{P(i,j|\theta)}{i^2}}{\sum_{i,j} P(i,j|\theta)}$$

High Gray Level Run Emphasis (HGLRE)

$$HGLRE = \frac{\sum_{i,j} i^2 P(i,j|\theta)}{\sum_{i,j} P(i,j|\theta)}$$

Short Run Low Gray Level Emphasis (SRLGLE)

$$SRLGLE = \frac{\sum_{i,j} \frac{P(i,j|\theta)}{i^2 j^2}}{\sum_{i,j} P(i,j|\theta)}$$

Short Run High Gray Level Emphasis (SRHGLE)

$$SRHGLE = \frac{\sum_{i,j} \frac{i^2 P(i,j|\theta)}{j^2}}{\sum_{i,j} P(i,j|\theta)}$$

Long Run Low Gray Level Emphasis (LRLGLE)

$$LRLGLE = \frac{\sum_{i,j} \frac{j^2 P(i,j|\theta)}{i^2}}{\sum_{i,j} P(i,j|\theta)}$$

Long Run High Gray Level Emphasis (LRHGLE)

$$LRHGLE = \frac{\sum_{i,j} i^2 j^2 P(i,j|\theta)}{\sum_{i,j} P(i,j|\theta)}$$

where $P(i,j|\theta)$ represented the gray level run length matrix. There were generally four calculation directions θ, including 0-degree, 45-degree, 90-degree, and 135-degree. N_p denoted the number of elements in the matrix.

3. Supplementary figures

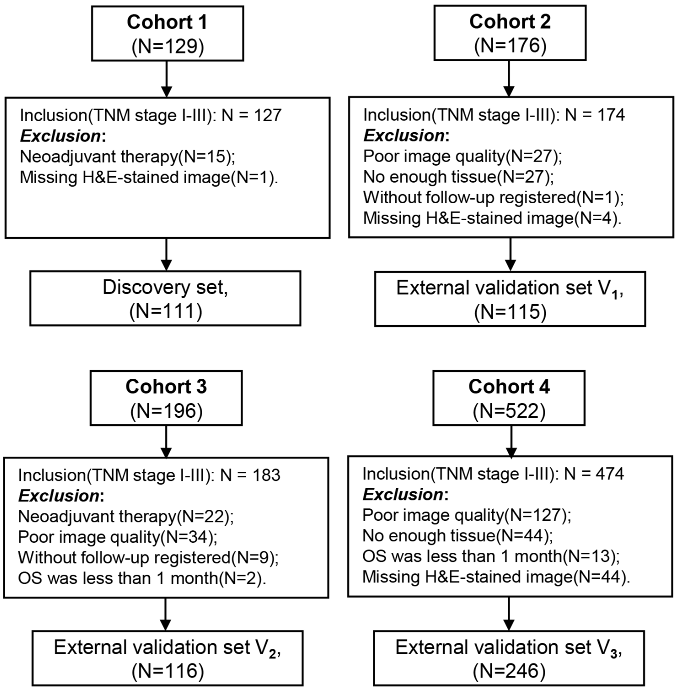

Figure S1. Data preparation and demographics of all cohorts.

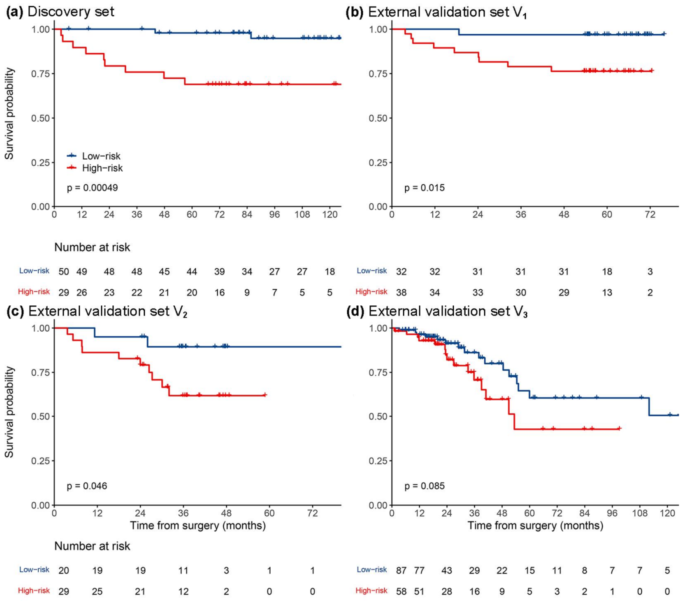

Figure S2. Kaplan–Meier curves of patients stratified by MPIS in the subgroup of patients with TNM stage I LUAD in the (**a**) discovery set; (**b**) external validation set V_1; (**c**) external validation set V_2; (**d**) external validation set V_3.

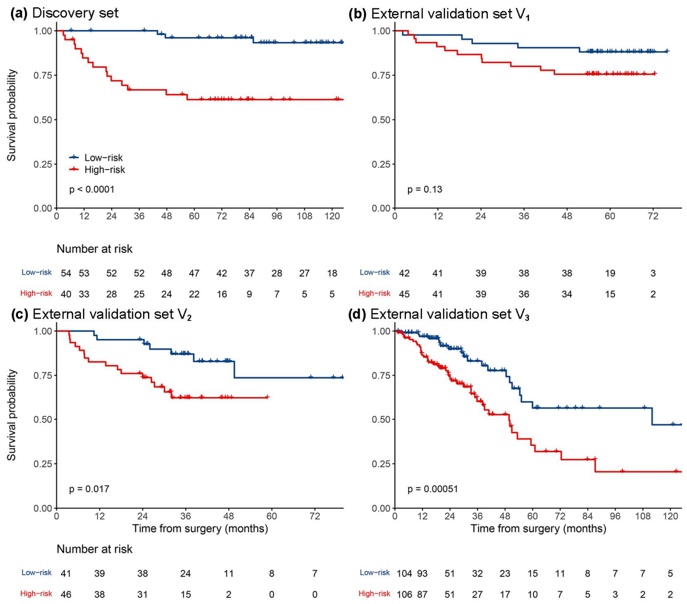

Figure S3. Kaplan–Meier curves of patients stratified by MPIS in the subgroup of patients with early-stage LUAD in the (**a**) discovery set; (**b**) external validation set V_1; (**c**) external validation set V_2; (**d**) external validation set V_3.

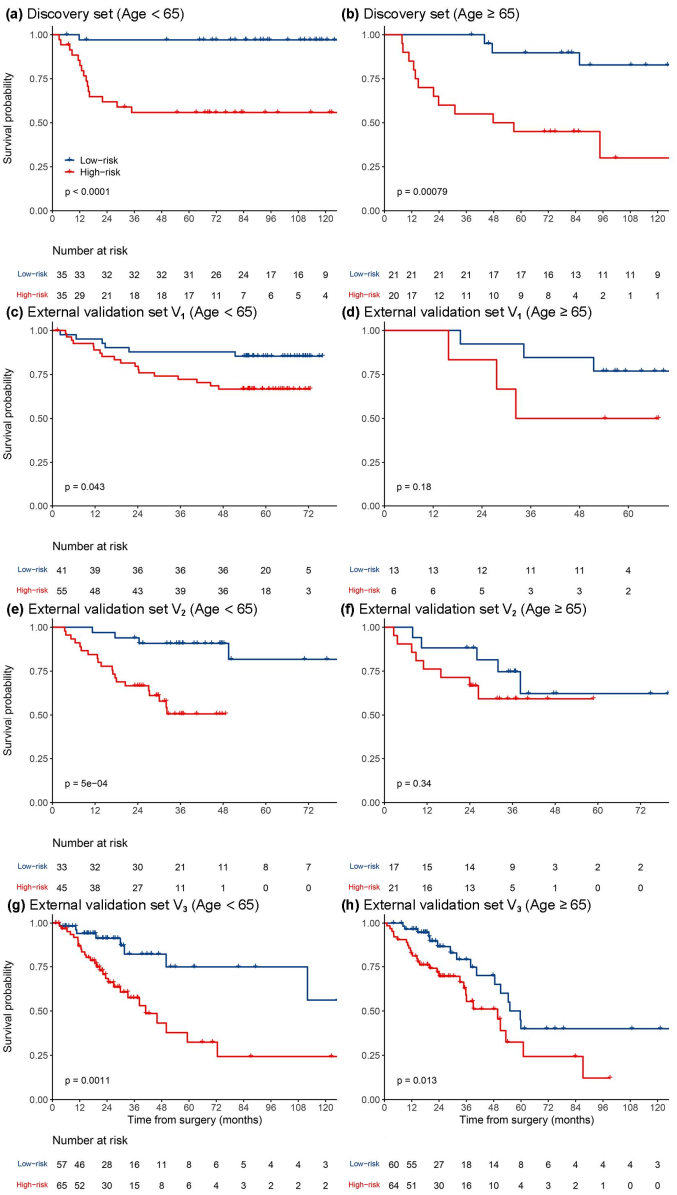

Figure S4. Kaplan–Meier curves of patients stratified by MPIS in the subgroups: (**a**) patients with age<65 years in the discovery set; (**b**) patients with age≥65 years in the discovery set; (**c**) patients with age<65 years in the external validation set V_1; (**d**) patients with age≥65 years in the external validation set V_1; (**e**) patients with age<65 years in the external validation set V_2; (**f**) patients with age≥65 years in the external validation set V_2; (**g**) patients with age<65 years in the external validation set V_3; (**h**) patients with age≥65 years in the external validation set V_3.

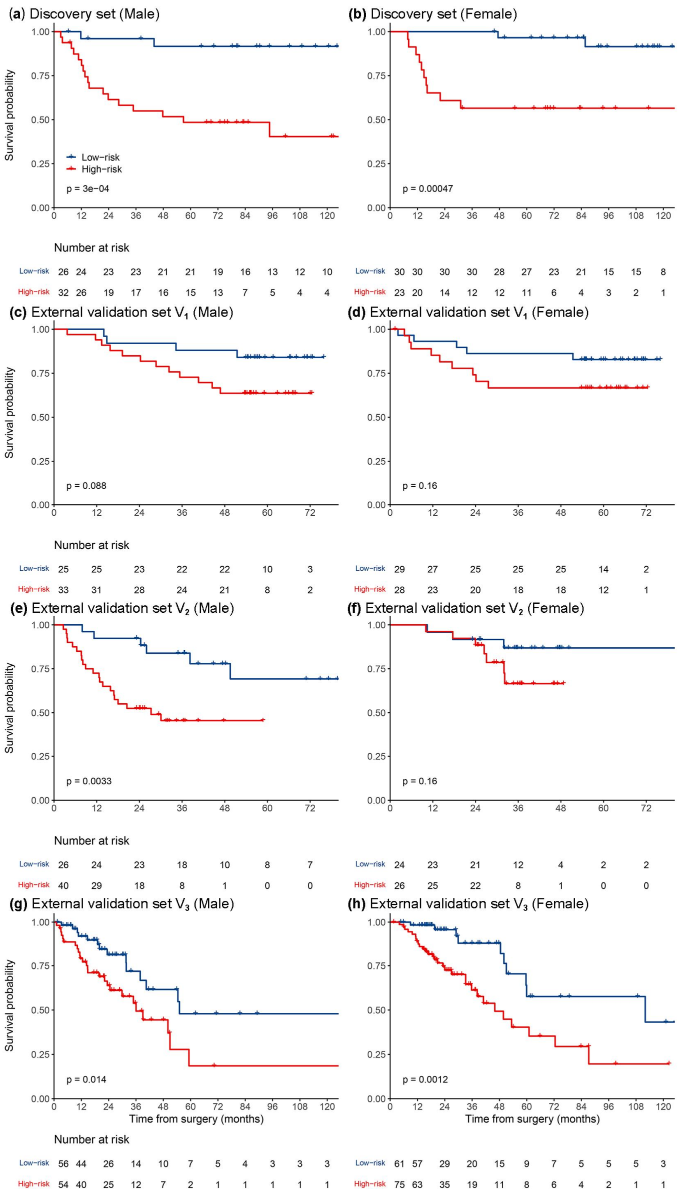

Figure S5. Kaplan–Meier curves of patients stratified by MPIS in the subgroups: (**a**) male sex in the discovery set; (**b**) female sex in the discovery set; (**c**) male sex in the external validation set V_1; (**d**) female sex in the external validation set V_1; (**e**) male sex in the external validation set V_2; (**f**) female sex in the external validation set V_2; (**g**) male sex in the external validation set V_3; (**h**) female sex in the external validation set V_3.

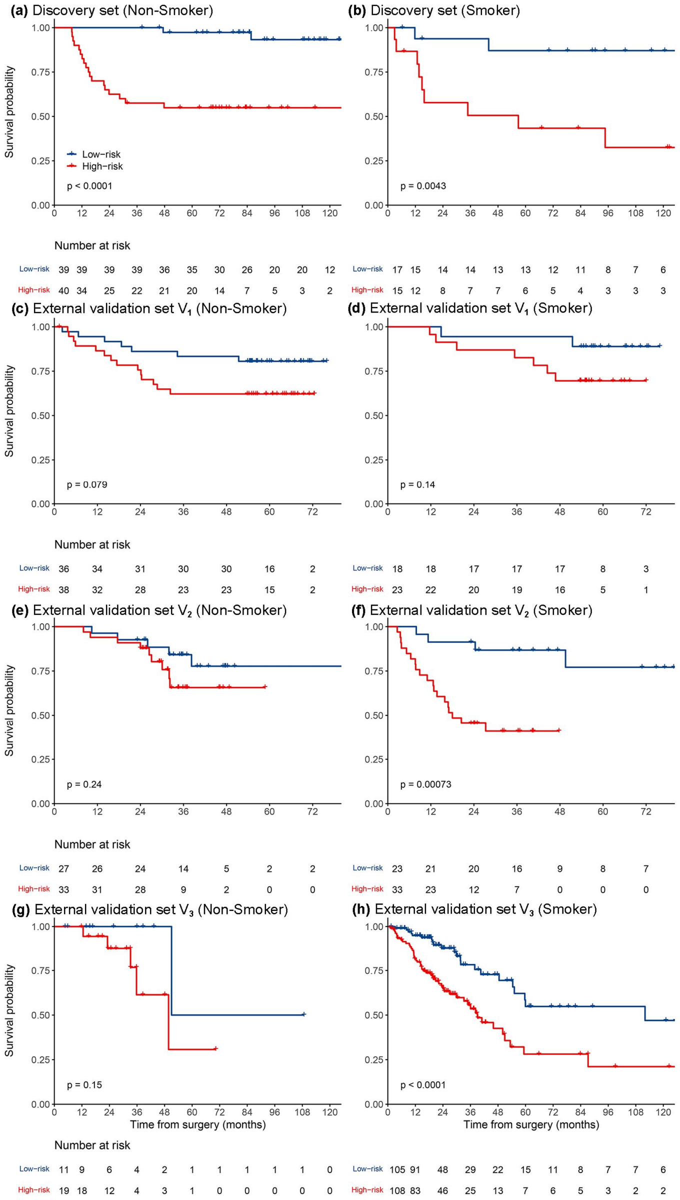

Figure S6. Kaplan–Meier curves of patients stratified by MPIS in the subgroups: (**a**) non-smoker in the discovery set; (**b**) smoker in the discovery set; (**c**) non-smoker in the external validation set V_1; (**d**) smoker in the external validation set V_1; (**e**) non-smoker in the external validation set V_2; (**f**) smoker in the external validation set V_2; (**g**) non-smoker in the external validation set V_3; (**h**) smoker in the external validation set V_3.

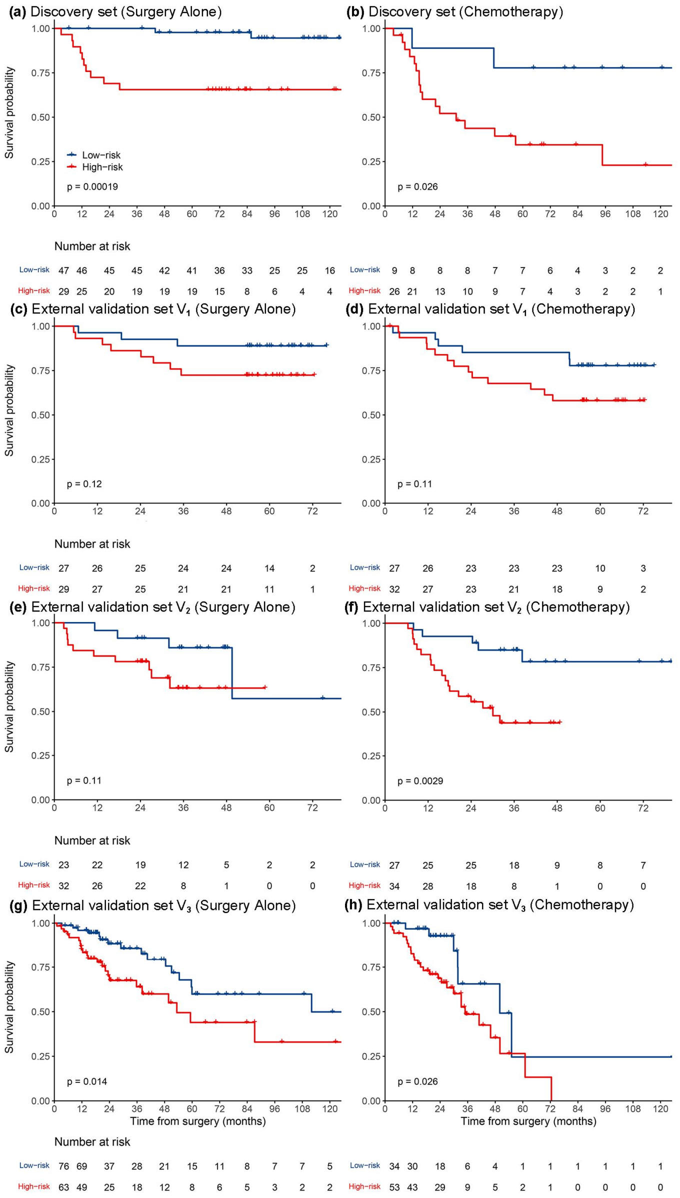

Figure S7. Kaplan–Meier curves of patients stratified by MPIS in the subgroups: (**a**) patients without adjuvant chemotherapy in the discovery set; (**b**) patients received adjuvant chemotherapy in the discovery set; (**c**) patients without adjuvant chemotherapy in the external validation set V_1; (**d**) patients received adjuvant chemotherapy in the external validation set V_1; (**e**) patients without adjuvant chemotherapy in the external validation set V_2; (**f**) patients received adjuvant chemotherapy in the external validation set V_2; (**g**) patients without adjuvant chemotherapy in the external validation set V_3; (**h**) patients received adjuvant chemotherapy in the external validation set V_3.

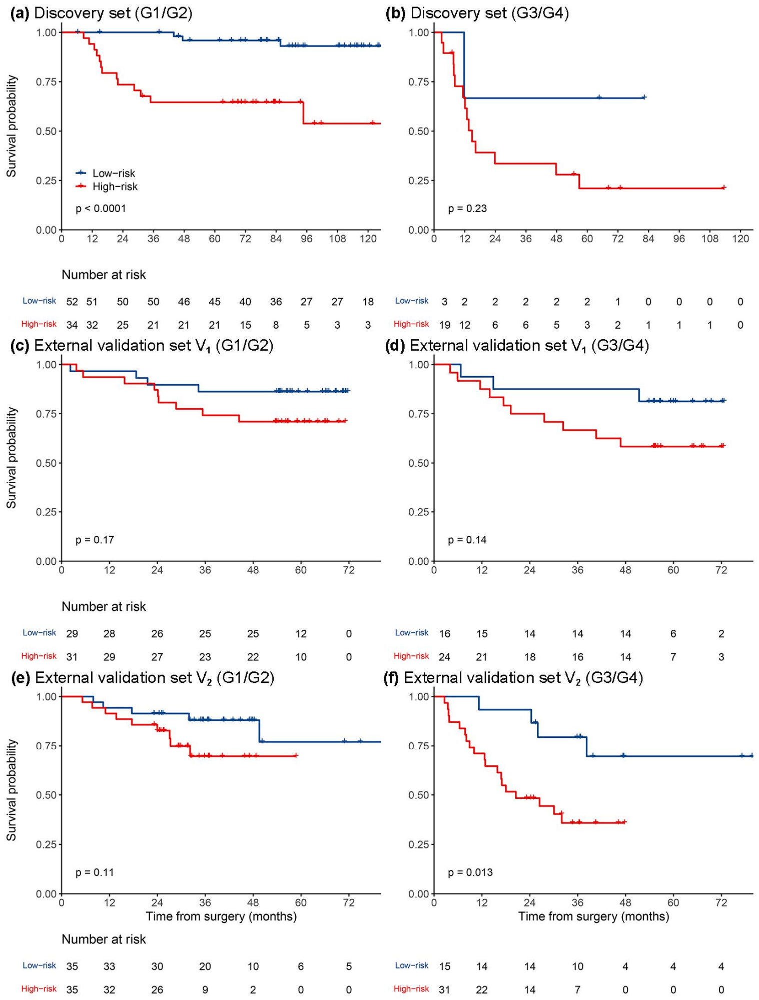

Figure S8. Kaplan–Meier curves of patients stratified by MPIS in the subgroups: (**a**) patients with well-moderately differentiated cancer in the discovery set; (**b**) patients with poorly-undifferentiated cancer in the discovery set; (**c**) patients with well-moderately differentiated cancer in the external validation set V_1; (**d**) patients with poorly-undifferentiated cancer in the external validation set V_1; (**e**) patients with well-moderately differentiated cancer in the external validation set V_2; (**f**) patients with poorly-undifferentiated cancer in the external validation set V_2.

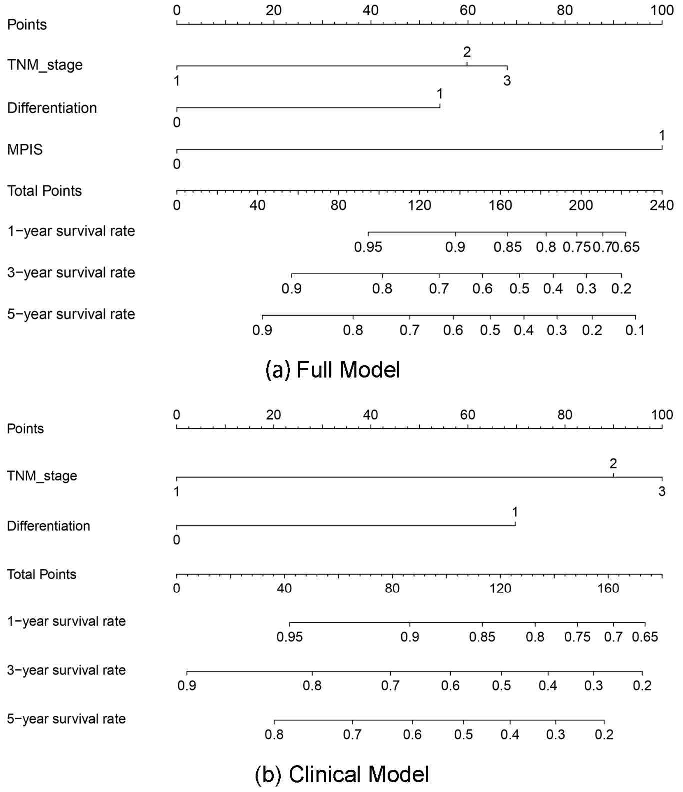

Figure S9. The visualization of the full model (**a**) and clinical model (**b**) as nomograms for patients with resectable LUAD.

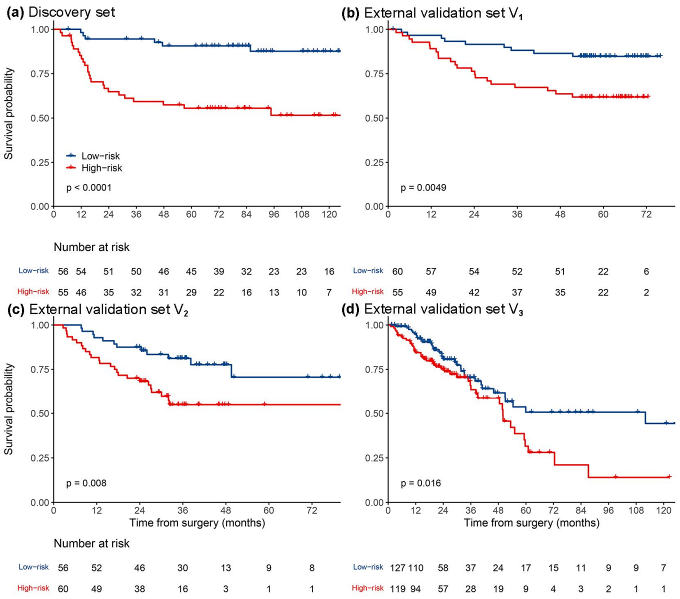

Figure S10. Kaplan–Meier curves of patients stratified by single-scale pathology image signature at 2.5× magnification in the (**a**) discovery set, (**b**) external validation set V_1, (**c**) external validation set V_2, and (**d**) external validation set V_3.

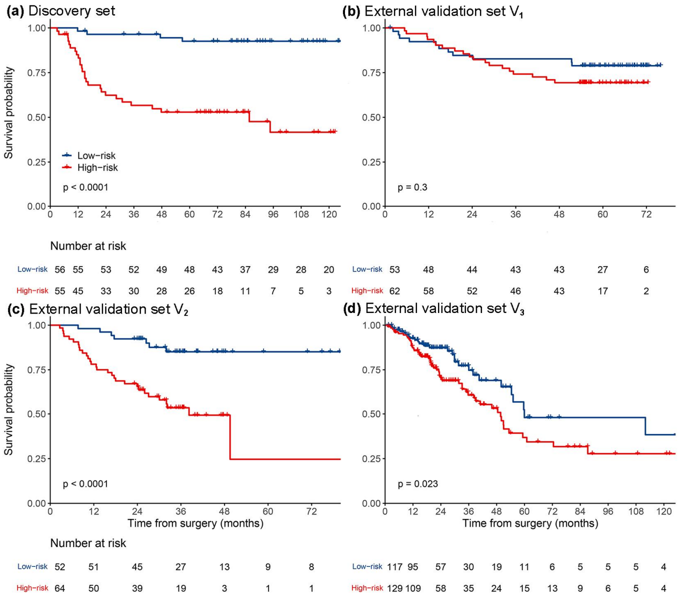

Figure S11. Kaplan–Meier curves of patients stratified by single-scale pathology image signature at 10× magnification in the (**a**) discovery set, (**b**) external validation set V_1, (**c**) external validation set V_2, and (**d**) external validation set V_3.

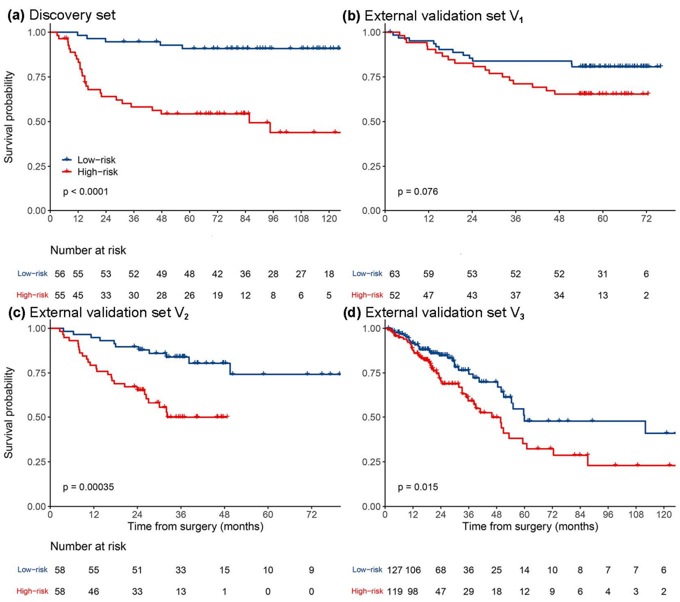

Figure S12. Kaplan–Meier curves of patients stratified by single-scale pathology image signature at 40× magnification in the (**a**) discovery set, (**b**) external validation set V_1, (**c**) external validation set V_2, and (**d**) external validation set V_3.

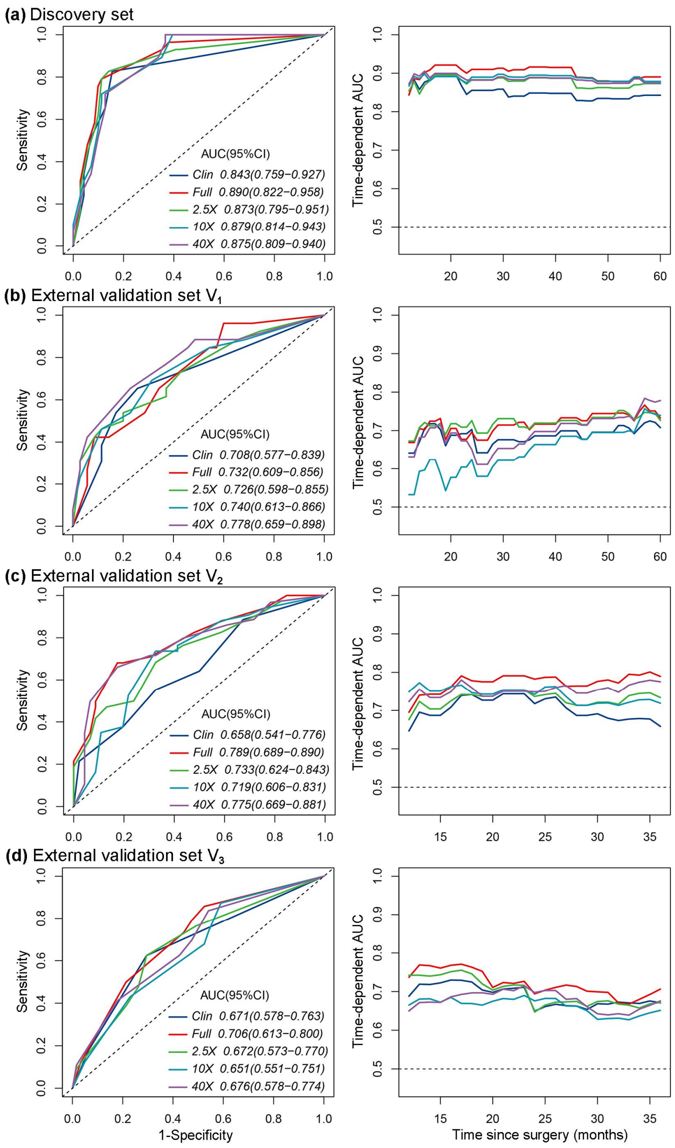

Figure S13. Time-dependent ROC curves and AUC curves of models in the (**a**) discovery set, (**b**) external validation set V_1, (**c**) external validation set V_2, and (**d**) external validation set V_3. Time-dependent ROC curves are evaluated for 5-year OS (or for 3-year OS), and time-dependent AUC curves are plotted for 12 to 60 months (or 12 to 36 months).

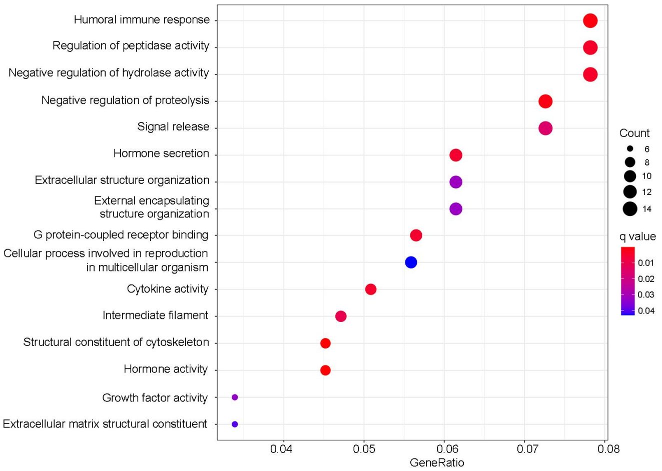

Figure S14. Significantly enriched biological pathways in Gene Ontology (GO) enrichment analysis.

4. Supplementary tables

Table S1. The specific definitions of the selected texture features.

Feature	Magnification	Definition
glrlm_SRLGLE_90_2.5	2.5×	The short run low gray level emphasis (SRLGLE) of GLRLM in a 90-degree direction.
glrlm_SRLGLE_90_40	40×	The short run low gray level emphasis (SRLGLE) of GLRLM in a 90-degree direction.
glcm_dissimilarity_2.5	2.5×	Dissimilarity of GLCM.
Kurtosis_10	10×	Kurtosis of the grayscale values.
glrlm_LRHGLE_90_2.5	2.5×	The long run high gray level emphasis (LRHGLE) of GLRLM in a 90-degree direction.
glrlm_SRE_0_40	40×	The short run emphasis (SRE) of GLRLM in a 90-degree direction.
glcm_ASM_2.5	2.5×	Angular second moment (ASM) of GLCM.
Percentile_10th_40	40×	The 10th percentile of the grayscale values.

Abbreviations: GLRLM, gray level run length matrix; GLCM, grey level co-occurrence matrix.

Table S2. The LASSO Cox selected features and corresponding coefficients to construct the MPIS.

Feature	Coefficient
glrlm_SRLGLE_90_2.5	-0.420951292
glrlm_SRLGLE_90_40	-0.604133682
glcm_dissimilarity_2.5	-0.305400033
Kurtosis_10	-0.385107632
glrlm_LRHGLE_90_2.5	0.522071813
glrlm_SRE_0_40	-0.01502991
glcm_ASM_2.5	0.275726346
Percentile_10th_40	0.538150006

Abbreviations: LASSO, least absolute shrinkage selection operator.

Table S3. The LASSO Cox selected features and corresponding coefficients to construct the single-scale pathology image signature at 2.5× magnification.

2.5× magnification	
**Feature**	**Coefficient**
glcm_dissimilarity	-0.489443909
glrlm_LRHGLE_0	0.839081469
glrlm_RP_90	0.059384676
glrlm_SRLGLE_90	-0.416953234

Abbreviations: LASSO, least absolute shrinkage selection operator.

Table S4. The LASSO Cox selected features and corresponding coefficients to construct the single-scale pathology image signature at 10× magnification.

10× magnification	
**Feature**	**Coefficient**
glcm_correlation	0.023625325
glrlm_SRLGLE_0	-0.730282591
percentile_10th	0.30181533
glrlm_RLN_135	0.501821261
Variance	0.051113712
glrlm_RLN_45	0.384302891

Abbreviations: LASSO, least absolute shrinkage selection operator.

Table S5. The LASSO Cox selected features and corresponding coefficients to construct the single-scale pathology image signature at 40× magnification.

40× magnification	
**Feature**	**Coefficient**
glrlm_LGLRE_0	-2.185308282
glrlm_SRE_0	0.269478825
percentile_10th	0.442351347
Kurtosis	-0.511713491
Maximum	0.569001482
glrlm_SRLGLE_90	1.905184662

Abbreviations: LASSO, least absolute shrinkage selection operator.
